# Time-Domain Characterization of Linear Viscoelastic Behavior in Asphalt Mixtures: A Comparative Evaluation Through Discrete and Continuous Spectral Techniques

**DOI:** 10.3390/polym17101299

**Published:** 2025-05-09

**Authors:** Fei Zhang, Bingyuan Huo, Wanmei Gui, Chao Li, Heng Liu, Yongming Xing, Lan Wang, Pucun Bai

**Affiliations:** 1 School of Materials Science and Engineering, Inner Mongolia University of Technology, Hohhot 010051, China; zhangfei@imut.edu.cn; 2 School of Civil Engineering, Inner Mongolia University of Technology, Hohhot 010051, China; h1606864536@163.com (B.H.); nmggydx@21cn.com (C.L.); 3 Inner Mongolia Autonomous Region Key Laboratory of Green Construction and Intelligent Operation and Maintenance of Civil Engineering, Hohhot 010051, China; xym@imut.edu.cn; 4 School of Architecture and Engineering, Yunnan Agricultural University, Kunming 650500, China; otherday475@gmail.com; 5 Inner Mongolia Autonomous Region Institute of Transportation Science and Development, Hohhot 010051, China; 15147136841@163.com

**Keywords:** road engineering, linear viscoelasticity, generalized sigmoidal model, relaxation time spectra, retardation time spectra, relaxation modulus, creep compliance

## Abstract

This study systematically investigates continuous and discrete spectra methodologies for determining time-domain viscoelastic response functions (creep compliance and relaxation modulus) in asphalt mixtures. Through complex modulus testing of three asphalt mixtures (base asphalt mixture, SBS-modified asphalt mixture, and crumb rubber-modified asphalt mixture), we established unified master curves using a Generalized Sigmoidal model with approximated Kramers–Kronig (K-K) relations. Discrete spectra can be obtained by Prony series of Maxwell/Kelvin modeling, while continuous spectra derived through integral transformation produced complementary response functions by numerical integration. Comparative analysis demonstrated that discrete and continuous spectra methods yield highly consistent predictions of the relaxation modulus and creep compliance within conventional time scales (10^−7^–10^5^ s), with significant deviations emerging only at extreme temporal extremities. Compared to discrete spectra results, material parameters (relaxation modulus and creep compliance) derived from continuous spectra methods invariably asymptotically approach upper and lower plateaus. Notably, the maximum equilibrium values derived from continuous spectra methods consistently surpassed those obtained through discrete approaches, whereas the corresponding minimum values were consistently lower. This comparative analysis highlights the inherent limitations in the extrapolation reliability of computational methodologies, particularly regarding spectra method implementation. Furthermore, within the linear viscoelastic range, the crumb rubber-modified asphalt mixtures exhibited superior low-temperature cracking resistance, whereas the SBS-modified asphalt mixtures demonstrated enhanced high-temperature deformation resistance. This systematic comparative study not only establishes a critical theoretical foundation for the precise characterization of asphalt mixture viscoelasticity across practical engineering time scales through optimal spectral method selection, but also provides actionable guidance for region-specific material selection strategies.

## 1. Introduction

Linear viscoelasticity represents a fundamental mechanical response characteristic of asphalt mixtures under combined low-temperature and high-frequency loading conditions. In the linear viscoelastic range, there is an intrinsic connection between the various viscoelastic response functions, that is, any viscoelastic response function can be obtained by transforming other types of response functions. The stresses and strains of linear viscoelastic materials always show a linear relationship at any time. Usually, viscoelastic materials behave as linear viscoelastic or nonlinear viscoelastic in different conditions [1], but linear viscoelasticity is the theoretical basis for the study of nonlinear viscoelasticity. Direct characterization of viscoelastic response functions in the time domain necessitates extended experimental durations due to inherent time-dependent material behaviors. Conversely, derivation of the relaxation modulus and creep compliance in the time domain through mathematical transformation of frequency-domain functions presents distinct advantages, including reduced experimental duration and enhanced measurement precision. As elucidated by the Boltzmann superposition principle, the complete viscoelastic characterization of materials can be fundamentally described through the evolution of relaxation (or retardation) time spectra [2], which quantitatively represent the time distribution of molecular motion modes within the material system. The material functions measured through diverse linear viscoelastic testing methodologies inherently share identical relaxation (or retardation) time spectra, constituting the fundamental framework and core principle governing all viscoelastic materials. These time spectra serve as a robust analytical method for characterizing the viscoelastic behavior of asphalt and asphalt mixtures [3], while simultaneously representing the most comprehensive frequency-dependent function in viscoelasticity. Substantiated by multiple research investigations [4,5], relaxation (or retardation) spectra have been established as intrinsic properties of linear viscoelastic materials. Significantly, the complete set of time-domain response functions for any linear viscoelastic material can be systematically derived from its corresponding spectral curves through rigorous mathematical formulations. According to the function form of the spectral intensity and the internal time of viscoelastic materials, it can be divided into discrete and continuous spectra, where the discrete spectra consist of a series of discrete spectral intensities with internal time, while the continuous spectra construct the functional relationship between the spectral intensity and the internal time.

To obtain discrete time spectra for asphalt materials, Tschoegl [6] describes a method for determining discrete relaxation time distributions from smoothed storage or loss modulus data, or discrete retardation time distributions from smoothed storage or loss compliance data. These time distributions can generate any other response curve. Zhao [7] et al. present a method for determining the coefficients of the generalized Maxwell (GM) model and generalized Kelvin (GK) model using the collocation method. The experimental results are presmoothed using the complex modulus master curve represented by the modified Havriliak–Negami (MHN) model, and then the model coefficients are solved from the storage or loss component collocation points. The GM and GK models obtained by this method are equivalent. The method provides a consistent and simple way to determine the GM and GK model coefficients for asphalt concrete. Finally, the GM and GK model coefficients are the discrete relaxation and discrete retardation spectra.

Considering the problems with discrete spectra, the continuous spectra concept has been gradually developed in recent years. The continuous spectra can be considered as discrete spectra with an infinitely dense time interval [8]. The studies of Mun [9] and Bhattacharjee [10] et al. showed that the continuous relaxation and retardation time spectra of asphalt mixtures can be obtained directly from the linear viscoelastic master curve model. The proposed analytical methodology demonstrates superior accuracy in deriving continuous relaxation spectra compared to conventional numerical discretization approaches, while maintaining direct applicability in the constitutive modeling of viscoelastic material behavior. To address the problem that the relaxation modulus obtained from the conventional Prony series model always accompanied negative model parameters and local oscillations of the curve, Luo [11] constructed the model describing the relaxation modulus in the form of Prony series with continuous relaxation spectra based on the creep test results, and the results showed that the model not only ensures positive parameters but also shows good fit. Zhao et al. [12] developed a new method to evaluate the effect of regeneration using relaxation spectra calculated from the dynamic shear rheological test data of asphalt binders; the results showed that the index developed from the relaxation spectra was able to assess the reduction in the proportion of large microstructures in aged asphalt binders, and the effectiveness of this method was demonstrated by scanning tunneling electron microscopy (STEM). To address the current drawbacks of the empirical and time-consuming selection of time coefficients in the Prony gradation, Lv et al. [13] proposed a method to determine the optimal relaxation time range based on the continuous relaxation spectra method and validated it using two different asphalt mixture complex modulus data. To study the effect of aging on the molecular weight and size of asphalt binders from dynamic mechanical test results, Yu et al. [14] analyzed the relaxation time spectra of three asphalt binders before and after aging by using the Generalized Sigmoidal function, and the results showed that the width of the relaxation time spectra of asphalt binders increased after aging. In addition, the zero-shear viscosity (ZSV) and activation energy of the binder can be accurately calculated by the relaxation time spectra. Zhang et al. [15] proposed a method to quickly obtain high-quality Prony series parameters of the relaxation modulus and creep compliance of asphalt concrete using complex modulus test data. The method employs continuous relaxation and retardation spectra from the Havriliak–Negami model and the 2S2P1D complex modulus model so that the discrete spectra can be directly determined and the relaxation modulus and compliance functions can be further calculated. The results show that the Havriliak–Negami and 2S2P1D models produce slightly different continuous spectral patterns for shorter relaxation times and longer retardation times. However, the continuum spectra of the two complex modulus models are very close to each other in the region covered by the test data. Therefore, the two models can generate comparable Prony sequence parameters over the time or frequency range covered by the test data. Zeng et al. [16] investigated the interconversion between the complex modulus, relaxation modulus, and creep compliance based on the collocation, multidata, and windowing methods, respectively, and showed that the windowing method caused fewer solution problems; however, when a narrower range of relaxation/retardation time is used, the collocation and multidata methods occasionally give more reasonable results. In addition, based on the strengths and weaknesses of the computational methods, a hybrid procedure, which takes advantage of each interconversion method, is proposed in this study to optimize the discrete spectrum determination for asphalt concrete. Han et al. [17] presented a method to study the response of the time-domain linear viscoelastic parameters of asphalt mixtures with the addition of different warm mix agents. The method utilizes the Generalized Sigmoidal function to construct the master curves of the storage and loss modulus in the frequency domain and uses discrete and continuous spectral to analyze the viscoelastic behavior of asphalt mixtures. The results show that the addition of the foam warm mix agent significantly reduces the relaxation modulus of the asphalt mixture by about 44%, while Sasobit and Evotherm slightly increase it by 14% and 22%, respectively. The foam warm mix also increased the equilibrium modulus of the creep compliance to 0.091 MPa, which is 80% higher than that of HMA. Lvet al. [18] developed a continuum spectral method to establish the interconversion between GM and GK model parameters. In the newly developed method, the complex modulus master curve is first constructed by means of a conventional master curve model. Continuous relaxation and retardation spectra are then built using the proposed direct or indirect methods. Subsequently, the time constants and strength constants of the GM and GK models are calculated by the extended search method, based on which the Prony series parameters and linear viscoelasticity variables of the asphalt mixtures are finalized. The successful application of this method to the Generalized Sigmoidal model demonstrates the versatility of the continuous spectrum method in determining the linear viscoelasticity variables of asphalt mixtures. Li et al. [19] investigated the use of dynamic shear rheological tests to analyze the viscoelastic properties of various fiber-reinforced asphalt binders. Using the dynamic modulus and phase angle data from the measured results, a generalized Maxwell model was used to select the appropriate elements and fit the test curves to the discrete time spectra according to the time–temperature equivalence principle. From this, master curves of the relaxation modulus and creep compliance were established to predict the relaxation and creep properties of various asphalt binders. The results show that the fiber-reinforced binder has greater resistance to high temperature and long-term deformation, as well as lower sensitivity to temperature and more significant elastic characteristics. On the other hand, based on the experimental data and the corresponding discussion, the 13-element GM model seems to be more suitable for fitting the data. In order to analyze the differences between the relaxation modulus master curves E(t) and creep compliance master curves J(t) obtained from the discrete and continuous spectral models, and to comprehensively evaluate the effect of the basalt fiber content on the viscoelastic behavior of asphalt mixtures, Huang et al. [20] conducted complex modulus tests on asphalt mixtures with a fiber content of 0%, 0.1%, 0.2%, and 0.3%, respectively. The results show that the addition of basalt fibers improves the strength, stress relaxation, and deformation resistance of asphalt mixtures.

In summary, asphalt mixtures exhibit pronounced viscoelastic behavior under varying frequency and temperature conditions. Existing research demonstrates that spectra-based approaches for obtaining time-domain relaxation and creep functions have attracted considerable attention, yet four critical limitations persist: (1) predominant focus on the interconversion of viscoelastic material functions rather than in-depth characterization of relaxation/retardation time spectra; (2) isolated investigations of either continuous or discrete time spectra without comparative analysis; (3) insufficient attention to retardation time spectra despite extensive studies on relaxation time spectra; and (4) limited exploration of discrete/continuous relaxation/retardation time spectra combinations, with existing studies prioritizing parametric influences over fundamental spectral properties. To address these limitations, this study investigates a base asphalt mixture (BM), SBS-modified asphalt mixture (SMM), and crumb rubber-modified asphalt mixture (RMM), deriving their discrete/continuous relaxation and retardation spectra through theoretical formulations based on complex modulus data from uniaxial loading tests, thereby enabling the comprehensive evaluation of their relaxation and creep characteristics across extended time domains. Specifically, the discrete relaxation and retardation spectra are analytically obtained from frequency-domain master curves using the generalized Maxwell and Kelvin models, respectively, while the continuous spectra are derived through integral transform theory applied to experimental master curves. This methodology offers dual advantages: eliminating the requirements for complex numerical optimization by directly extracting spectra parameters from dynamic test data and enabling the precise reconstruction of time-domain relaxation modulus and creep compliances through both discrete and continuous spectral representations. Notably, the inherent convenience of constructing frequency-domain master curves from complex modulus measurements significantly streamlines spectra determination. The technical significance of this research manifests in two aspects: fundamentally, the acquired discrete/continuous spectra serve as critical constitutive parameters for characterizing asphalt mixture viscoelasticity, providing essential inputs for material modeling; and practically, the established spectra analysis framework offers novel theoretical tools for mixture design optimization, performance prediction, and pavement structural enhancement. Through comparative analysis of the spectral characteristics across three asphalt mixtures, this work elucidates the mechanistic impacts of different modification technologies on time–temperature dependencies, ultimately advancing theoretical foundations for developing functional pavement materials with tailored viscoelastic performance.

## 2. Materials and Methods

### 2.1. Materials

Three types of asphalt binders (base asphalt, SBS-modified asphalt, and crumb rubber-modified asphalt) were combined with mineral aggregates to fabricate AC-16 asphalt mixtures through the Marshall design methodology for experimental investigation, with the resulting specimens designated as BM, SMM, and RMM, respectively. The Panjin 90# asphalt was used as the base asphalt for the experimental study; the SBS-modified asphalt containing 4% linear styrene–butadiene–styrene (SBS) copolymer (by mass of base asphalt) was procured from Inner Mongolia Transportation Materials Co., Ltd. (Hohhot, China) The rubber-modified asphalt was produced under controlled laboratory conditions utilizing a standardized protocol: 40-mesh bias tire rubber powder (constituting 20% *w*/*w* of base asphalt mass) was blended with Panjin 90# base asphalt in an open-blade mechanical mixer at 180 ± 2 °C for 30 min under continuous shearing at 700 rpm. Based on the rigorous testing protocols outlined in the ‘Standard Test Methods for Bitumen and Bituminous Mixtures in Highway Engineering’ (JTG E20-2011) [21], the performance parameters of the asphalt binder were verified to fully comply with the specified technical requirements of the standard. Comprehensive evaluation confirmed that all critical properties, including but not limited to penetration grade, softening point, ductility, and aging resistance characteristics, demonstrated satisfactory conformity with the prescribed acceptance criteria for highway pavement applications. The respective gradations of coarse aggregate, fine aggregate, and mineral filler employed in the experimental investigation demonstrated full compliance with the prescribed specification requirements. Finally, the volume parameters of the three kinds of mixture are shown in Table 1. A rigorous triplicate sampling protocol was implemented to ensure measurement reliability, with three independent replicate tests conducted for each mixture and a statistical control criterion enforced requiring all experimental measurements to remain within 1.15 standard deviations of the mean.

### 2.2. Methods

#### 2.2.1. Test Method for Dynamic Modulus

The dynamic modulus test was employed to investigate the linear viscoelastic behavior of asphalt mixtures through comprehensive experimental characterization. The standard test specimen size was Φ100 mm × H150 mm, and the test was performed across varying temperature and loading frequencies. In order to better control the shape of the master curve, the test was conducted at eleven temperatures of −20 °C, −10 °C, 5 °C, 10 °C, 20 °C, 25 °C, 35 °C, 45 °C, 50 °C, 55 °C, and 60 °C, and each temperature was also tested at a frequency of 25 Hz, 10 Hz, 5 Hz, 1 Hz, 0.5 Hz, and 0.1 Hz. The test started at the lowest temperature (−20 °C) and terminated at the highest temperature (60 °C). And each specimen was tested with a frequency sweep from the highest frequency (25 Hz) to the lowest frequency (0.1 Hz), with an interval of 2 min between any two frequencies. To maintain testing within the linear viscoelastic range, the specimen strain must be controlled between 50 με and 115 με (AASHTO TP 79-15 [22]). The load–deformation hysteresis curves from the final five loading cycles are recorded for subsequent computation of the dynamic modulus and phase angle, where the dynamic modulus represents the stiffness of the material under cyclic loading and the phase angle represents quantifying the time-dependent viscous response relative to the elastic behavior. The calculation equations for complex modulus $E^{*}$, dynamic modulus $\left|E^{*}\right|$, and phase angle φ are provided in Equations (A1)–(A3) in Appendix A.1.

#### 2.2.2. Time–Temperature Superposition Principle

This investigation addresses the critical challenge of characterizing asphalt pavement behavior across extreme operational thermal gradients (construction temperatures: 80–180 °C; service conditions: −30–60 °C) and complex loading regimes combining high-speed traffic impulses with prolonged thermal–mechanical relaxation effects. While conventional laboratory testing cannot practically replicate this full spectrum of environmental and mechanical demands, the time–temperature superposition principle (TTSP) enables comprehensive viscoelastic modeling through master curve construction. The developed methodology imposes two fundamental constraints: (1) strict adherence to K-K relations [23] governing inter-parameter consistency between storage/loss components, (2) universal shift factor applicability across all viscoelastic functions. A Generalized Sigmoidal model framework incorporating approximate K-K relations was developed to establish a robust characterization methodology for the intrinsic asymmetric viscoelastic behavior of asphalt materials. The implemented K-K approximation enforces the required causal relationship between real and imaginary response components—a critical physical constraint unattainable through conventional analytical approaches.

Both the TTSP and K-K relations are the important components of linear viscoelastic theory. The TTSP is the mathematical conversion rule that quantitatively describes the time–temperature equivalence effect of viscoelastic materials, and the K-K relations are a relationship that must be satisfied between the real part and the imaginary part of the viscoelastic material. Within the framework of linear viscoelastic theory, the constitutive relationships between key viscoelastic parameters—specifically the storage modulus $E^{\prime }$ and loss modulus $E^{\prime \prime }$, as well as their compliance counterparts (storage compliance $D^{\prime }$ and loss compliance $D^{\prime \prime }$)—must rigorously satisfy the K-K relations. The TTSP facilitates the determination of shift factors through three established methodologies: the Arrhenius equation [24], Williams–Landel–Ferry (WLF) equation [25], and quadratic polynomial equation [26,27] can be used to calculate the shift factor. Given that the Arrhenius formula is difficult to characterize the shift factor at higher temperatures, and the quadratic polynomial equation lacks sufficient theoretical basis, the WLF formula is used for the calculation of the shift factor, and its form is given in the following Equation (1).

(1) $$\mathrm{lg}\alpha_{T}=\frac{-C_{1}\left(T-T_{r}\right)}{C_{2}+\left(T-T_{r}\right)}$$

where $\alpha_{T}$ denotes the shift factor; $C_{1}$, $C_{2}$ denote the fitting constants of WLF; *T_r_* and *T* denote the reference temperature and test temperature, respectively, °C.

#### 2.2.3. Method for Constructing Master Curves of Storage Modulus and Loss Modulus Based on the K-K Relations

The storage modulus $E^{\prime }$ and loss modulus $E^{\prime \prime }$ are derived from experimentally measured dynamic modulus $\left|E^{*}\right|$ and phase angle φ data through the linear viscoelastic constitutive relationships defined in Equations (A4) and (A5) in Appendix A.2. The master curve development process employs the following: (1) the Generalized Sigmoidal function to characterize the evolution of the storage modulus; (2) approximate K-K relations to establish the $E^{\prime }$-$E^{\prime \prime }$ relationship [28]; and finally, the functional forms of the master curve of the storage modulus and the loss modulus are shown in Equations (2) and (3), where the reference temperature $T_{r}=25\;{}^{\circ}C$.

(2) $$\mathrm{lg}\left|E^{\prime }\left(f_{r}\right)\right|=\delta^{\prime }+\frac{\alpha^{\prime }}{{\left(1+\lambda^{\prime }e^{\beta^{\prime }+\gamma^{\prime }\mathrm{lg}\left(f_{r}\right)}\right)}^{\frac{1}{\lambda^{\prime }}}}$$

(3) $$E^{\prime \prime }\left(f_{r}\right)=\frac{\pi }{2}k^{\prime }E^{\prime }\left(f_{r}\right)\frac{d\left(\mathrm{lg}\left|E^{\prime }\right|\right)}{d\left(\mathrm{lg}f_{r}\right)}=-\frac{\pi }{2}E^{\prime }\left(f_{r}\right)k^{\prime }\alpha^{\prime }\gamma^{\prime }\frac{e^{\beta^{\prime }+\gamma^{\prime }\mathrm{lg}f_{r}}}{{\left(1+\lambda^{\prime }e^{\beta^{\prime }+\gamma^{\prime }\mathrm{lg}f_{r}}\right)}^{1+\frac{1}{\lambda^{\prime }}}}$$

where $lg\left|E^{\prime }\left(f_{r}\right)\right|$ denotes the logarithm of the storage modulus, MPa; $\delta^{\prime }$ denotes the lower asymptotic value of the storage modulus master curve; $\alpha^{\prime }$ denotes the vertical distance between the upper and lower asymptotes of the storage modulus master curve; $\beta^{\prime }$ and $\gamma^{\prime }$ denote the shape coefficients of the storage modulus master curve, where $\gamma^{\prime }$ governs the transition rate between asymptotic plateaus, with larger values inducing sharper transitions; $\beta^{\prime }$ significantly influences the horizontal position of the transition point; $\lambda^{\prime }$ determines the asymmetric characteristics of the storage modulus master curve model, and $\lambda^{\prime }=1$ is the symmetric Sigmoidal model, $\lambda^{\prime }\neq 1$ is the asymmetric Generalized logarithmic Sigmoidal model; $E^{\prime \prime }\left(f_{r}\right)$ denotes a modified functional form of the loss modulus master curve obtained from the Generalized Sigmoidal storage modulus master curve model based on the K-K relations; $k^{\prime }$ is the auxiliary fitting parameter of the loss modulus.

#### 2.2.4. Method for Constructing Master Curves of Storage Compliance and Loss Compliance Based on the K-K Relations

Based on the mathematical relationships established in Equations (A6) and (A7) in Appendix A.3, the storage compliance $D^{\prime }$ and loss compliance $D^{\prime \prime }$ can be mathematically determined through viscoelastic interconversion formulae using experimentally obtained storage modulus $E^{\prime }$ and loss modulus $E^{\prime \prime }$ datasets. This analytical procedure enables precise transformation between complementary viscoelastic functions while maintaining thermodynamic consistency throughout the conversion process. Following an analogous procedure to the modulus characterization, a Generalized Sigmoidal model was used to mathematically describe the storage compliance master curve for the reference temperature $T_{r}=25\;{}^{\circ}C$, with its analytical expression explicitly defined in Equation (4). Through application of the approximate K-K relations that establish the causal interdependence between storage compliance and loss compliance, the mathematical formulation of the frequency-dependent loss compliance master curve was systematically derived via analytic continuation techniques, as formally expressed in Equation (5). This derivation procedure strictly adheres to the principle of causality embedded in linear viscoelasticity theory, ensuring the resultant spectral representation maintains self-consistent phase relationships across the entire frequency domain. The final mathematical formulations governing both master curves are systematically expressed through Equations (4) and (5), respectively, thereby completing the comprehensive analytical framework for viscoelastic compliance characterization. This dual-equation system enables precise reconstruction of compliance behavior across extended time scales while maintaining thermodynamic consistency between the storage and loss components.

(4) $$\mathrm{lg}\left|D^{\prime }\left(f_{r}\right)\right|=\delta^{\prime \prime }+\frac{\alpha^{\prime \prime }}{{\left(1+\lambda^{\prime \prime }e^{\beta^{\prime \prime }+\gamma^{\prime \prime }\mathrm{lg}\left(f_{r}\right)}\right)}^{\frac{1}{\lambda^{\prime \prime }}}}$$

(5) $$D^{\prime \prime }\left(f_{r}\right)=\frac{\pi }{2}k^{\prime \prime }D^{\prime }\left(f_{r}\right)\frac{d\left(\mathrm{lg}\left|D^{\prime }\left(f_{r}\right)\right|\right)}{d\left(\mathrm{lg}f_{r}\right)}=-\frac{\pi }{2}D^{\prime }\left(f_{r}\right)k^{\prime \prime }\alpha^{\prime \prime }\gamma^{\prime \prime }\frac{e^{\beta^{\prime \prime }+\gamma^{\prime \prime }\mathrm{lg}f_{r}}}{{\left(1+\lambda^{\prime \prime }e^{\beta^{\prime \prime }+\gamma^{\prime \prime }\mathrm{lg}f_{r}}\right)}^{1+\frac{1}{\lambda^{\prime \prime }}}}$$

where $lg\left|D^{\prime }\left(f_{r}\right)\right|$ denotes the logarithm of the storage compliance; $\delta^{\prime \prime }$ denotes the lower asymptotic value of the storage compliance master curve; $\alpha^{\prime \prime }$ denotes the vertical distance between the upper and lower asymptotes of the storage compliance master curve; $\beta^{\prime \prime }$ and $\gamma^{\prime \prime }$ are the shape parameters of the storage compliance master curve, where $\gamma^{\prime \prime }$ governs the transition rate between asymptotic plateaus, with larger values inducing sharper transitions; $\beta^{\prime \prime }$ governs the horizontal positioning of the inflection point along the reduced frequency axis; $\lambda^{\prime \prime }$ determines the asymmetric characteristics of the storage compliance master curve model, and $\lambda^{\prime \prime }=1$ is the symmetric Sigmoidal model, $\lambda^{\prime \prime }\neq 1$ is the asymmetric Generalized logarithmic Sigmoidal model; $D^{\prime \prime }\left(f_{r}\right)$ corresponds to the loss compliance function derived through K-K relations from the Generalized Sigmoidal storage compliance formulation; $k^{\prime \prime }$ serves as an auxiliary fitting parameter that optimizes the loss compliance master curve approximation while maintaining K-K consistency.

## 3. Results and Discussion

### 3.1. Discrete Time Spectra of Asphalt Mixtures

#### 3.1.1. Discrete Relaxation Time Spectra

The master curves for both the storage modulus and loss modulus in asphalt mixtures may be mathematically established through synergistic integration of the Generalized Sigmoidal constitutive model with approximate K-K relations. Using the WLF shift technique, the fitting parameters of the master curve model are calibrated by the least squares method considering the measured results of the storage modulus and loss modulus. Among them, the Generalized Sigmoidal constitutive model comprises two interrelated components: the storage modulus master curve and the loss modulus master curve. The storage modulus formulation incorporates five independent model parameters ($\delta^{\prime }$, $\alpha^{\prime }$, $\beta^{\prime }$, $\gamma^{\prime }$, $\lambda^{\prime }$), while the loss modulus expression shares four fundamental parameters ($\alpha^{\prime }$, $\beta^{\prime }$, $\gamma^{\prime }$, $\lambda^{\prime }$) with the storage modulus model, in addition to the necessary time–temperature shift factor parameter ($C_{1}$, $C_{2}$). Additionally, the loss modulus characterization requires a supplementary auxiliary fitting parameter ($k^{\prime }$). All the parameters in Equations (2) and (3) are solved simultaneously by constructing the error functions of the measured and model-predicted storage and loss modulus, and ensuring that the error function $ef_{1}$ is minimized. The form of the error function is given in Equation (6). The model parameters were determined through numerical optimization utilizing the solve function within Microsoft Excel’s computational framework. The derived parameter estimates are subsequently summarized in Table 2, thereby ensuring comprehensive presentation of the analytical outcomes. Table 2 shows that the maximum error $ef_{1}$ of the model is only 4.3%, indicating that the results are reliable.

(6) $$ef_{1}=ef_{E^{\prime }}+ef_{E^{\prime \prime }}=\frac{1}{N}\sqrt{\sum_{i=1}^{N}{\left(\frac{{\left|E^{\prime }\right|}_{m,i}-{\left|E^{\prime }\right|}_{p,i}}{{\left|E^{\prime }\right|}_{m,i}}\right)}^{2}}+\frac{1}{N}\sqrt{\sum_{i=1}^{N}{\left(\frac{{\left|E^{\prime \prime }\right|}_{m,i}-{\left|E^{\prime \prime }\right|}_{p,i}}{{\left|E^{\prime \prime }\right|}_{m,i}}\right)}^{2}}$$

where $ef_{1}$ denotes the error function quantifying the relative deviation between the experimental measurements and model predictions for both the storage modulus and loss modulus, %; $ef_{E^{\prime }}$ denotes the error function of the storage modulus, %; $ef_{E^{\prime \prime }}$ denotes the error function of the loss modulus, %; N is the test sample space of the dynamic modulus with the value of 66; ${\left|E^{\prime }\right|}_{m,i}$ denotes the storage modulus further calculated from the complex modulus test, MPa; ${\left|E^{\prime }\right|}_{p,i}$ denotes the storage modulus predicted from the Generalized Sigmoidal model, MPa; ${\left|E^{\prime \prime }\right|}_{m,i}$ denotes the loss modulus obtained by further calculation from the complex modulus test, MPa; ${\left|E^{\prime \prime }\right|}_{p,i}$ denotes the loss modulus predicted from the Generalized Sigmoidal model and combined with the K-K relations, MPa.

**Figure 1:**
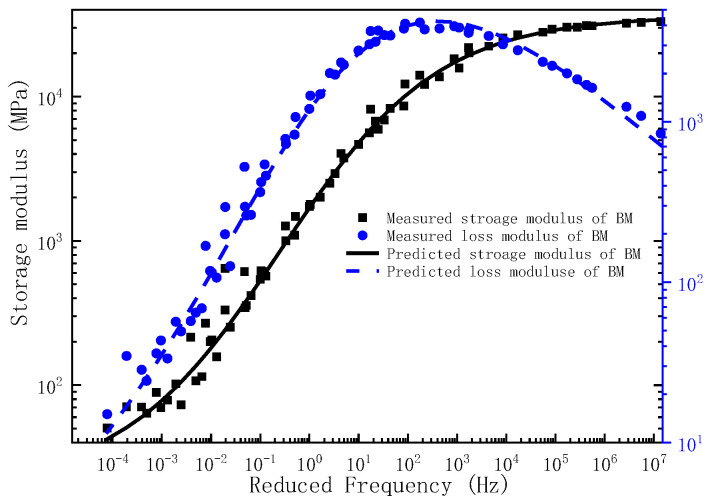
The master curve of storage modulus and loss modulus for BM.

**Figure 2:**
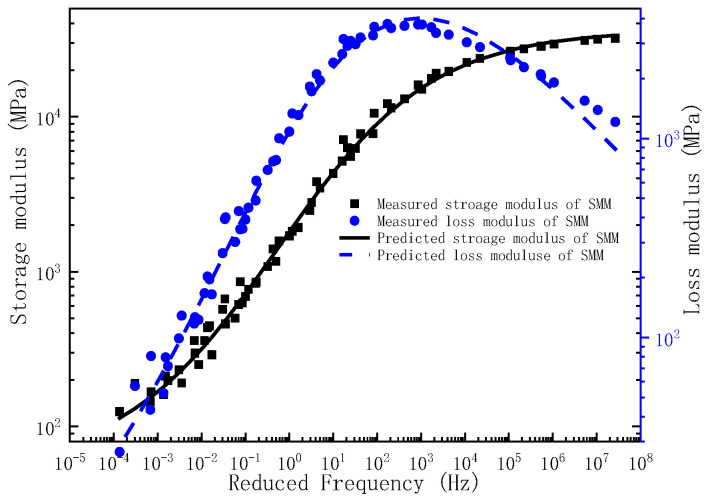
The master curve of storage modulus and loss modulus for SMM.

**Figure 3:**
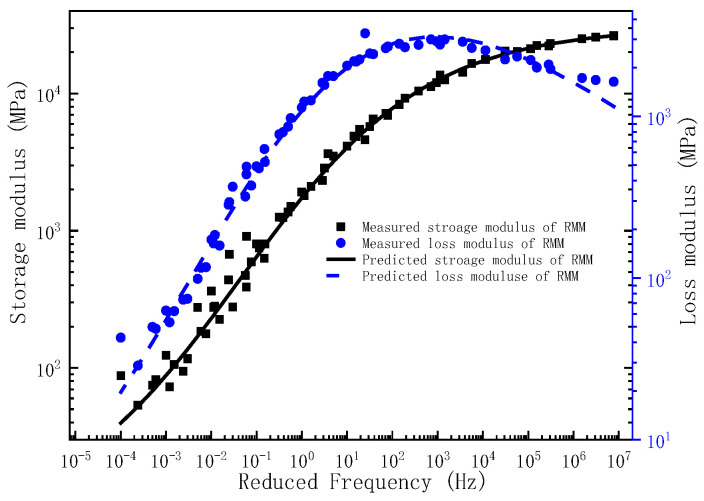
The master curve of storage modulus and loss modulus for RMM.

Upon determination of the governing parameters for the storage modulus and loss modulus master curves, the corresponding frequency–temperature-dependent master curves can be analytically derived. To validate the predictive capacity of the proposed model, the experimentally measured storage modulus and loss modulus across the full frequency are comparatively plotted against the model predictions in Figure 1, Figure 2 and Figure 3. As evidenced by Figure 1, Figure 2 and Figure 3, the experimental measurements and model predictions of the storage modulus and loss modulus for the three asphalt mixtures (BM, SMM, RMM) exhibit significant alignment, thereby validating the reliability of the optimized parameters listed in Table 2 for subsequent time-domain spectral characterization, including discrete relaxation spectrum derivation.

The generalized Maxwell model [29] has been extensively adopted for characterizing the time-dependent relaxation response of asphalt mixtures within their linear viscoelastic range, with its mathematical representation taking the form of a Prony series expansion as detailed in Equation (7).

(7) $$E\left(t\right)=E_{e}+\sum_{i=1}^{N}E_{i}e^{-\frac{t}{\rho_{i}}}=E_{g}-\sum_{i=1}^{N}E_{i}(1-e^{-\frac{t}{\rho_{i}}})$$

where $E\left(t\right)$ denotes the relaxation modulus, MPa; $E_{e}$ denotes the equilibrium modulus, MPa; $E_{g}$ denotes the glassy modulus, MPa; $E_{i}$ denotes the relaxation strength, MPa; and $\rho_{i}$ denotes the discrete relaxation time, s.

The discrete relaxation time spectrum comprises a set of distinct relaxation times and their associated modulus coefficients, which collectively define the viscoelastic response characteristics of the material [30]. To numerically reconstruct this discrete relaxation time spectrum, the storage modulus and loss modulus derived from Equations (2) and (3) are rewritten into Prony series representations, as respectively expressed in Equations (8) and (9). Then, the error function $ef_{2}$ is formulated to quantify the magnitude of discrepancies between the Prony series approximation and the viscoelastic modulus derived from the Generalized Sigmoidal model. The form of the error function $ef_{2}$ is shown in the following Equation (10). By solving Equations (8) and (9) simultaneously using the nonlinear least squares optimization and ensuring that the error function $ef_{2}$ is minimal, the coefficients of the Prony series for each mixture are determined and the discrete relaxation time spectra are further obtained. Finally, the discrete relaxation spectra of all the mixtures can be found, and the results are shown in Table 3. Table 4 shows that the maximum error $ef_{2}$ of the model is only 4.9%, indicating that the results are reliable.

(8) $$E^{\prime }\left(w\right)=E_{e}+\sum_{i=1}^{m}\frac{w^{2}{\rho_{i}}^{2}{E_{i}}^{2}}{w^{2}{\rho_{i}}^{2}+1}$$

(9) $$E^{\prime \prime }\left(w\right)=\sum_{i=1}^{m}\frac{w\rho_{i}E_{i}}{w^{2}{\rho_{i}}^{2}+1}$$

where $E^{\prime }\left(w\right)$ and $E^{\prime \prime }\left(w\right)$ are the storage modulus and loss modulus obtained from the Generalized Sigmoidal model and approximate K-K relations; $w=2\pi f_{r}$ denotes the reduced angular frequency, which can be used to express the angular frequency at the test temperature relative to that of the reference temperature, rad/s.

(10) $$ef_{2}=ef_{E_{ps}^{\prime }}+ef_{E_{ps}^{\prime \prime }}=\frac{1}{N}\sqrt{\sum_{i=1}^{N}{\left(\frac{{\left|E^{\prime }\right|}_{ps,i}-{\left|E^{\prime }\right|}_{gsm,i}}{{\left|E^{\prime }\right|}_{gsm,i}}\right)}^{2}}+\frac{1}{N}\sqrt{\sum_{i=1}^{N}{\left(\frac{{\left|E^{\prime \prime }\right|}_{ps,i}-{\left|E^{\prime \prime }\right|}_{gsm,i}}{{\left|E^{\prime \prime }\right|}_{gsm,i}}\right)}^{2}}$$

where ${ef}_{2}$ denotes the error function that quantifies the discrepancy between the storage modulus and loss modulus obtained through Prony series approximation and those characterized by the Generalized Sigmoidal model, %; N is the fitted data point, which is also taken as 66 in here; ${\left|E^{\prime }\right|}_{gsm,i}$ denotes the computationally predicted storage modulus derived through parametric implementation of the Generalized Sigmoidal constitutive model, MPa; and ${\left|E^{\prime }\right|}_{ps,i}$ denotes the predicted storage modulus based on the Prony series, MPa. ${\left|E^{\prime \prime }\right|}_{gsm,i}$ denotes the computationally predicted loss modulus derived through parametric implementation of the Generalized Sigmoidal constitutive model and the approximate K-K relations, MPa; and ${\left|E^{\prime \prime }\right|}_{ps,i}$ denotes the predicted loss modulus based on the Prony series, MPa.

#### 3.1.2. Discrete Retardation Time Spectra

The master curves of the storage compliance and loss compliance for the asphalt mixtures were also developed using the Generalized Sigmoidal model coupled with approximate K-K relations. A comprehensive parameter determination approach was implemented through nonlinear least squares optimization, wherein the WLF equation was systematically applied to achieve horizontal shifting of the viscoelastic data. This calibration methodology simultaneously incorporated experimental measurements of both the storage and loss compliance components to ensure thermorheologically consistent master curve construction. The integrated optimization framework enabled the concurrent determination of the sigmoidal model coefficients and WLF shift factors while maintaining physical consistency between the real and imaginary compliance responses through K-K compatibility constraints. The Generalized Sigmoidal storage compliance master curve model incorporates five distinct fitting parameters ($\delta^{\prime \prime }$, $\alpha^{\prime \prime }$, $\beta^{\prime \prime }$, $\gamma^{\prime \prime }$, $\lambda^{\prime \prime }$), while its corresponding loss compliance master curve model shares four common parameters ($\alpha^{\prime \prime }$, $\beta^{\prime \prime }$, $\gamma^{\prime \prime }$, $\lambda^{\prime \prime }$) with the storage compliance and introduces an additional auxiliary parameter ($k^{\prime \prime }$) specific to its characterization. The results of the shift factor parameter ($C_{1}$, $C_{2}$) are consistent with the previous results, and the reduced frequency is directly used here to characterize them. The error function $ef_{3}$ of the storage and loss compliance is constructed as well to ensure the value is minimum; moreover, all model parameters are obtained by solving Equations (4) and (5) simultaneously. The form of the error function is given in Equation (11). All model parameters, including both the model fitting parameters and the shift factor parameters, were determined through numerical optimization using the solver function in Microsoft Excel. The derived parameter values, obtained through this computational approach, are comprehensively presented in Table 5, which summarizes the final quantitative results of the analytical procedure. Table 5 shows that the maximum error $ef_{3}$ of the model is only 5.6%, indicating that the results are reliable.

(11) $$ef_{3}=ef_{D^{\prime }}+ef_{D^{\prime \prime }}=\frac{1}{N}\sqrt{\sum_{i=1}^{N}{\left(\frac{{\left|D^{\prime }\right|}_{m,i}-{\left|D^{\prime }\right|}_{p,i}}{{\left|D^{\prime }\right|}_{m,i}}\right)}^{2}}+\frac{1}{N}\sqrt{\sum_{i=1}^{N}{\left(\frac{{\left|D^{\prime \prime }\right|}_{m,i}-{\left|D^{\prime \prime }\right|}_{p,i}}{{\left|D^{\prime \prime }\right|}_{m,i}}\right)}^{2}}$$

where $ef_{3}$ denotes the error function of the storage and loss compliance, %; $ef_{D^{\prime }}$ denotes the error function of the storage compliance, %; $ef_{D^{\prime \prime }}$ denotes the error function of the loss compliance, %; N is the fitted data point, which is also taken as 66 in here; ${\left|D^{\prime }\right|}_{m,i}$ denotes the storage compliance obtained by further calculation from the complex modulus test, MPa^−1^; ${\left|D^{\prime }\right|}_{p,i}$ denotes the storage compliance predicted from the Generalized Sigmoidal model, MPa^−1^; ${\left|D^{\prime \prime }\right|}_{m,i}$ denotes the loss compliance obtained by further calculation from the complex modulus test, MPa^−1^; and ${\left|D^{\prime \prime }\right|}_{p,i}$ denotes the loss compliance predicted from the Generalized Sigmoidal model combined with the approximate K-K relations, MPa^−1^.

**Figure 4:**
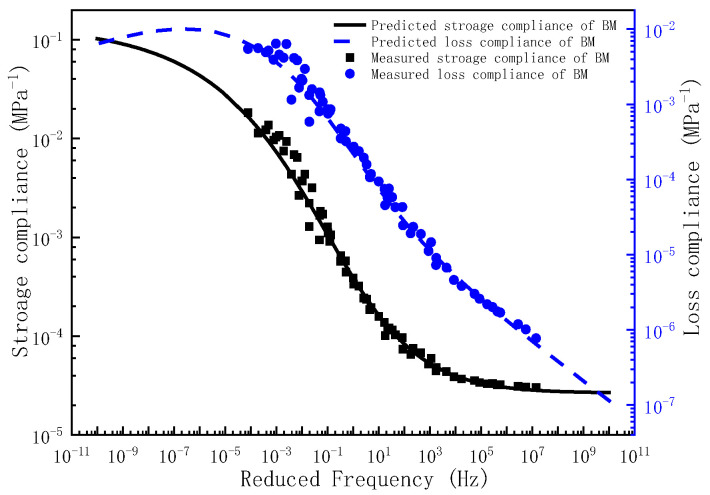
The master curve of storage compliance and loss compliance for BM.

**Figure 5:**
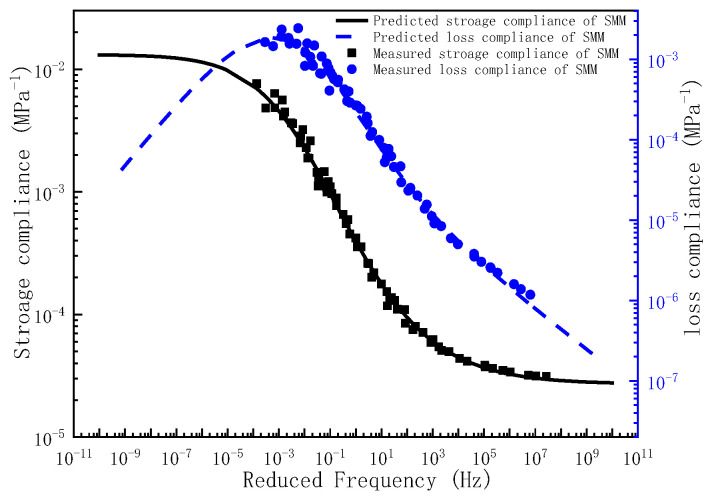
The master curve of storage compliance and loss compliance for SMM.

**Figure 6:**
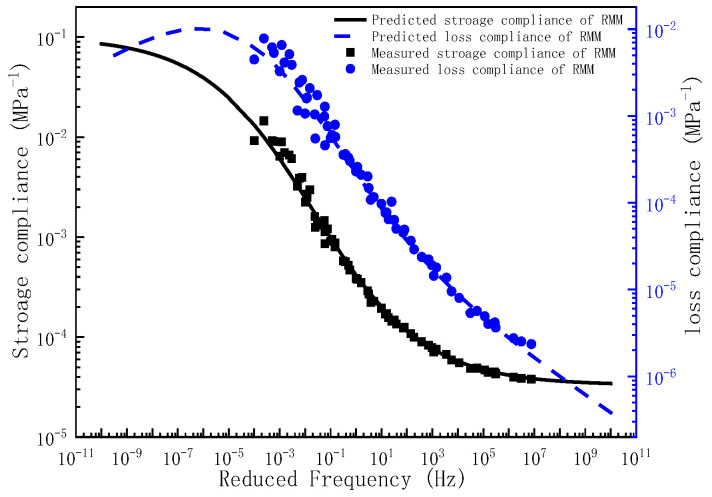
The master curve of storage compliance and loss compliance for RMM.

Upon determination of the governing parameters for the storage compliance and loss compliance master curves, the corresponding frequency–temperature-dependent master curves can be analytically derived. To validate the predictive capacity of the proposed model, the experimentally measured storage compliance and loss compliance across the full frequency are comparatively plotted against the compliance predictions in Figure 4, Figure 5 and Figure 6. As evidenced by Figure 4, Figure 5 and Figure 6, the experimental measurements and model predictions of the storage compliance and loss compliance for the three asphalt mixtures exhibit significant alignment, thereby validating the reliability of the optimized parameters listed in Table 5 for the subsequent time-domain spectral characterization, including discrete retardation spectrum resolution through inverse Laplace transformation.

The generalized Kelvin model [31] has been extensively employed to model the creep response of asphalt mixtures within their linear viscoelastic range. The mathematical representation of the creep compliance function in this constitutive model is formulated using a Prony series, as demonstrated in Equation (12).

(12) $$D\left(t\right)=D_{g}+\sum_{j=1}^{N}D_{j}\left(1-e^{-\frac{t}{\tau_{j}}}\right)=D_{e}-\sum_{j=1}^{N}D_{j}e^{-\frac{t}{\tau_{j}}}$$

where $D\left(t\right)$ denotes the creep compliance, MPa^−1^; $D_{g}$ denotes the glass compliance, MPa^−1^; $D_{j}$ denotes the retarded strength, MPa^−1^; $D_{e}=D_{g}+D_{j}$ denotes the long-term equilibrium compliance, MPa^−1^; and $\tau_{j}$ denotes the discrete retardation time, s.

The discrete retardation time spectrum comprises a set of discrete retardation times and their corresponding retardation strength values. To numerically reconstruct this spectrum, the storage compliance and loss compliance derived from Equations (4) and (5) are rewritten into Prony series representations, as respectively expressed in Equations (13) and (14). The error function $ef_{4}$ is then introduced to characterize the error between the Prony series and the Generalized Sigmoidal model in terms of the storage and loss compliance. The error function $ef_{4}$ is expressed by the form of Equation (15). Similar to the previous procedure, Equations (13) and (14) are solved simultaneously again using the least squares method to ensure that the error function $ef_{4}$ is minimized to find the Prony series coefficients for each type of mixture and further to obtain the discrete retardation time spectra. Finally, the discrete retardation time spectra of mixtures are shown in Table 6. As can be seen from the results in Table 7, the maximum error of the model is no more than 8%, indicating that the results are reliable.

(13) $$D^{\prime }\left(w\right)=D_{g}+\sum_{j=1}^{N}\frac{D_{j}}{w^{2}\tau_{j}^{2}+1}$$

(14) $$D^{\prime \prime }\left(w\right)=\frac{1}{\eta_{0}w}+\sum_{j=1}^{N}\frac{w\tau_{j}D_{j}}{w^{2}\tau_{j}^{2}+1}$$

where $D^{\prime }\left(w\right)$ and $D^{\prime \prime }\left(w\right)$ are the storage compliance and loss compliance obtained from the Generalized Sigmoidal model rewritten in the form of a Prony series.

(15) $$ef_{4}=ef_{D_{ps}^{\prime }}+ef_{D_{ps}^{\prime \prime }}=\frac{1}{N}\sqrt{\sum_{i=1}^{N}{\left(\frac{{\left|D^{\prime }\right|}_{ps,i}-{\left|D^{\prime }\right|}_{gsm,i}}{{\left|D^{\prime }\right|}_{gsm,i}}\right)}^{2}}+\frac{1}{N}\sqrt{\sum_{i=1}^{N}{\left(\frac{{\left|D^{\prime \prime }\right|}_{ps,i}-{\left|D^{\prime \prime }\right|}_{gsm,i}}{{\left|D^{\prime \prime }\right|}_{gsm,i}}\right)}^{2}}$$

where $ef_{4}$ denotes the error function of the storage compliance and loss compliance described based on the Prony series and the Generalized Sigmoidal model, %; N is the fitted data point, which is also taken as 66 in here; ${\left|D^{\prime }\right|}_{gsm,i}$ denotes the analytically derived storage compliance obtained through implementation of the Generalized Sigmoidal constitutive framework within its master curve formulation, MPa^−1;^ ${\left|D^{\prime }\right|}_{ps,i}$ denotes the storage compliance predicted by the Prony series form, MPa^−1^; ${\left|D^{\prime \prime }\right|}_{gsm,i}$ denotes the analytically derived loss compliance obtained through implementation of the Generalized Sigmoidal constitutive framework within its master curve formulation and the K-K relations, MPa^−1^; and ${\left|D^{\prime \prime }\right|}_{ps,i}$ denotes the loss compliance expressed through the Prony series, MPa^−1^.

#### 3.1.3. Study on Discrete Relaxation and Retardation Time Spectra of Different Asphalt Mixtures

**Figure 7:**
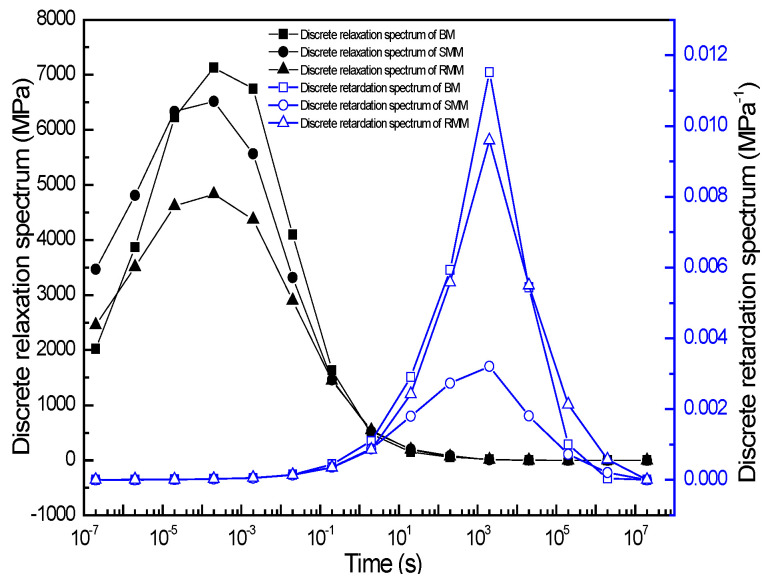
The discrete relaxation and retardation time spectra for three kinds of mixture.

For comparative analysis of the discrete relaxation and retardation spectra characterizing the different mixtures, the respective spectral data are plotted in a single figure to facilitate direct visual comparison. As illustrated in Figure 7, both spectral distributions exhibited distinct bell-shaped profiles with characteristic time domains. The relaxation spectrum demonstrated a pronounced peak (maximum intensity) centered at 10^−4^ s, with the BM exhibiting the highest peak value of 7130 MPa, followed by the SMM at 6516 MPa and RMM at 4830 MPa. In contrast, the retardation spectrum demonstrated a pronounced peak (maximum intensity) centered at 10^3^ s, with the BM achieving the maximum peak value of 1.2 × 10^−2^ MPa^−1^, followed by the RMM at 9.6 × 10^−3^ MPa^−1^ and SMM at 3.2 × 10^−3^ MPa^−1^. The peak point of the relaxation spectra in the figure is consistent with Gundla’s results [32]. When the relaxation time is greater than 10^−4^ s, the relaxation spectra intensity of the BM is the largest, followed by that of the SMM, and the relaxation spectra intensity of the RMM is the smallest. When the relaxation time was less than 10^−4^ s, the relaxation spectra intensity of the BM decreased rapidly until it was lower than the relaxation spectra intensity of the RMM. However, in all discrete relaxation time ranges, the relaxation spectra intensity and width of the SMM were always larger than those of the RMM, although there were also few data for the discrete relaxation time spectra at this time to determine the spectral intensity variation pattern at shorter relaxation times, which is the limit of the discrete relaxation time spectra. It is also known that when the retardation time is less than 10^3^ s, the retardation spectral intensity of the BM is the largest, followed by the RMM, and the retardation spectral intensity of the SMM is the smallest. When the retardation time was greater than 10^3^ s, the retardation spectra intensity of the BM decreased rapidly until it gradually approached the retardation spectra intensity of the SMM. However, in all discrete retardation time ranges, the retardation spectra intensity and width of the RMM were always larger than those of the SMM, although the retardation time spectra had too few data to accurately determine the retardation spectra intensity at longer retardation times, which is the limitation of the discrete retardation time spectra. From the discrete spectra, it can also be found that the width and intensity of the relaxation spectra of the modified asphalt mixture are smaller than the BM, indicating that the modified asphalt mixture has better stress relaxation ability; in addition, the retardation spectra intensity of the modified mixture also becomes smaller, and the retardation spectra intensity and width of the SMM are the smallest. It shows that the modified asphalt mixture has better deformation resistance than the BM, and the SMM has the best deformation resistance.

### 3.2. Continuous Time Spectra of Asphalt Mixture

Although discrete time spectra serve as a robust analytical tool for characterizing the viscoelastic behavior of asphalt mixtures, their practical application presents two inherent limitations. Primarily, the morphological features and spectral intensity distributions exhibit significant dependence on the selected discrete time intervals. Furthermore, the derived viscoelastic response functions tend to manifest oscillatory artifacts within localized time domains. These computational challenges can be effectively resolved through transition to continuous time spectral analysis. The continuous time spectral comprises two complementary components: the continuous relaxation time spectrum and the continuous retardation time spectrum, which, respectively, govern stress relaxation and creep recovery processes in viscoelastic materials.

#### 3.2.1. Continuous Relaxation Time Spectra

Within the framework of integral transform theory, the continuous spectra of relaxation times can be mathematically extracted from the frequency-domain representations of viscoelastic material functions. For practical applications, this study specifically presents the spectral distribution derived from the master curve representation of the storage modulus, as this parameter provides critical insights into the hierarchical relaxation processes of viscoelastic materials. The methodology preserves rigorous mathematical relationships between time-domain relaxation phenomena and frequency-dependent mechanical responses, while maintaining computational tractability for complex material systems. Equation (16) mathematically represents the continuous relaxation time spectrum, whose complete derivation adheres to the theoretical framework established by Tschoegl [18]. From the results of Tschoegl, it is clear that once the functional form of the storage modulus master curve model is known, the angular frequency *w* in the storage modulus master curve model is replaced by $\rho^{-1}\exp\left(\pm j\pi /2\right)$, and only the imaginary part of the master curve functional form is retained, and the functional form of the continuous relaxation time spectra $H\left(\rho \right)$ can be obtained by Equation (16).

(16) Hρ=±2πImE′ρ−1exp±jπ2

While Equation (16) inherently prevents the direct acquisition of continuous relaxation time spectra from the storage modulus master curves, this limitation is resolved through strategic mathematical transformation. By applying Euler’s formula combined with de Moivre’s theorem, the original formulation undergoes critical restructuring to yield Equation (17)—an optimized expression enabling efficient implementation. The complete derivation procedure, maintaining rigorous mathematical consistency throughout the transformation, is systematically documented in Appendix A.4 for verification and reproducibility.

(17) $$H\left(\rho \right)=-\left(\frac{2}{\pi }\right)\exp\left(A+X\left(\rho \right)\right)\sin\left(Y\left(\rho \right)\right)$$

#### 3.2.2. Continuous Retardation Time Spectra

Similar to the continuous relaxation time spectra, the continuous retardation time spectra can be derived from the functional form of viscoelastic response functions in the frequency domain. For methodological clarity, this presentation specifically demonstrates the continuous retardation time spectra characterized through the functional formalism of the storage compliance master curve. Equation (18) is the functional form for the continuous retardation time spectra, and the detailed derivation can be found in the results of Tschoegl [18]. According to the results of Tschoegl, it can be seen that once the functional form of the storage compliance master curve model is obtained, the angular frequency *w* in the storage compliance master curve model is replaced by $\tau^{-1}\exp\left(\pm j\pi /2\right)$, and only the imaginary part of the functional form of the storage compliance master curve is retained, and the functional form of the continuous retardation time spectra $L\left(\tau \right)$ can be deduced by the solution.

(18) Lτ=∓2πImD′τ−1exp±jπ2

Similar to the continuous relaxation time spectrum, while Equation (18) inherently prevents the direct acquisition of the continuous retardation time spectra from the storage compliance master curves, this limitation is resolved through strategic mathematical transformation. By applying Euler’s formula combined with de Moivre’s theorem, the original formulation undergoes critical restructuring to yield Equation (19)—an optimized expression enabling efficient implementation. The complete derivation procedure, maintaining rigorous mathematical consistency throughout the transformation, is systematically documented in Appendix A.5 for verification and reproducibility.

(19) $$L\left(\tau \right)=\left(\frac{2}{\pi }\right)\exp\left[A-X\left(\tau \right)\right]\sin\left[Y\left(\tau \right)\right]$$

#### 3.2.3. Study on Continuous Relaxation and Retardation Time Spectra of Different Asphalt Mixtures

**Figure 8:**
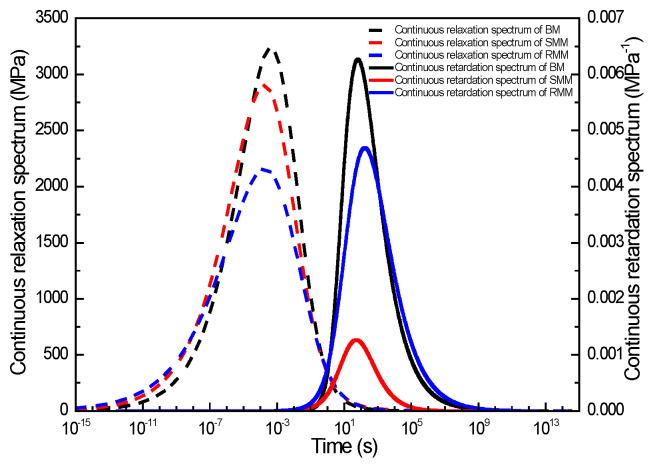
The continuous relaxation and retardation time spectra for three kinds of mixture.

To comparatively analyze the continuous relaxation spectra and continuous retardation spectra across the three asphalt mixtures, the spectral distributions of each mixture were co-plotted in a unified graphical representation. Figure 8 presents these continuous relaxation and retardation spectra profiles for the three asphalt mixtures, enabling direct visual comparison of their viscoelastic characteristics. As illustrated in Figure 8, similar to the discrete time spectra, both continuous spectral distributions exhibited distinct bell-shaped profiles with characteristic time domains. The relaxation spectrum demonstrated a pronounced peak (maximum intensity) centered at 10^−4^ s, with the BM exhibiting the highest peak value of 3230 MPa, followed by the SMM at 2899 MPa and RMM at 2155 MPa. In contrast, the continuous retardation spectrum demonstrated a pronounced peak (maximum intensity) centered at 10^3^ s, with the BM achieving the maximum peak value of 6.3 × 10^−3^ MPa^−1^, followed by the RMM at 4.7 × 10^−3^ MPa^−1^ and SMM at 1.3 × 10^−3^ MPa^−1^. In addition, it is also found that the asymmetric properties of the continuous retardation spectra are more significant than the continuous relaxation spectra, which is caused by the fact that the asymmetric properties of the storage modulus master curve are less than the storage compliance master curve. This finding further demonstrates the effectiveness of constructing master curves for viscoelastic response functions through the Generalized Sigmoidal model in conjunction with approximate K-K relations. Notably, when the relaxation time domain exceeds 10^−4^ s, the BM exhibits the highest continuous relaxation spectrum intensity. This is followed sequentially by the SMM, while the RMM consistently demonstrates the lowest spectral intensity across this time range. When the relaxation time was less than 10^−4^ s, the relaxation spectra of the rubber and SMM were close to each other and gradually tended to zero, while the continuous relaxation spectra of the BM decreased rapidly until lower than the rubber and SMM. Moreover, when the retardation time was less than 10^3^ s, the retardation spectra intensity of the BM was the largest, followed by the RMM, and the retardation spectra intensity of the SMM was the smallest. When the retardation time was greater than 10^3^ s, the retardation spectra intensity of the BM decreased rapidly until it was lower than that of the RMM, and the retardation spectra intensity of the SMM was always the smallest.

Comparing the continuous relaxation time spectra of the base and the two modified asphalt mixtures, it was found that the peak intensity (maximum relaxation intensity) of the relaxation spectra of the modified asphalt mixture was smaller than that of the BM and the width was larger than that of the BM, and the peak point of the relaxation spectra of the modified mixture shifted to the left horizontally. This suggests that compared to the BM, the modified asphalt mixture requires a shorter duration to complete the stress relaxation process. The accelerated relaxation rate observed in the modified mixture can be attributed to its reduced temperature susceptibility and lower glass transition temperature (T_g_) [33]. Notably, the test temperature of 25 °C significantly exceeds the T_g_ of the asphalt mixture [34], a condition under which the modified mixture demonstrates enhanced viscoelastic performance. The combination of these factors enables the modified asphalt to maintain superior viscoelastic behavior at this service temperature, exhibiting more efficient energy dissipation characteristics during the relaxation process. From a molecular perspective, the mechanism underlying these observations can be principally attributed to the formation of larger-scale molecular aggregates within the material matrix [35]. The observed broadening of the relaxation spectra manifests as a direct consequence of two synergistic factors: progressive molecular chain elongation accompanied by corresponding increases in molecular weight distribution, coupled with the strategic incorporation of polar functional groups that enhance interfacial interactions within the binder’s polymeric network [36]. Comparing the continuous retardation time spectra of the base and the two modified asphalt mixtures, it was found that the peak intensity and width of the retardation spectra of SMM were always smaller than those of the BM, while the peak intensity of the retardation spectra of the RMM was smaller than that of the BM and the width was comparable to that of the BM, which indicated that at longer times, the creep compliance of the SMM is the smallest, followed by the RMM, while the creep compliance of the BM is the largest. This conclusion is also consistent with the creep compliance results presented below.

### 3.3. Master Curve of Relaxation Modulus

Stress relaxation is the fundamental mechanical phenomenon of viscoelastic materials [37]. Stress relaxation is a phenomenon in which the stress gradually decreases with increasing time when the constant strain is applied. The relaxation modulus [38] is an important parameter to evaluate the relaxation capacity. In asphalt pavement design, the relaxation modulus serves as a critical parameter for stress prediction. However, direct experimental determination of this property imposes exceptional demands on instrumentation capabilities. To address this challenge, conventional methodologies typically derive storage modulus and loss modulus master curves from dynamic modulus test data. Subsequently, the Prony series formulation is employed to achieve simultaneous fitting of both storage and loss modulus master curves. This fitting process enables the determination of discrete relaxation spectra, from which the time-domain relaxation modulus master curve can be reconstructed. Alternatively, a continuous relaxation spectrum may be derived through integral inverse transformation of the functional representation of the storage modulus master curve. This continuous spectrum approach facilitates the computation of the relaxation modulus master curve via numerical integration techniques. Both methodologies provide theoretically consistent frameworks for converting frequency-domain viscoelastic characteristics to time-domain relaxation behavior, thereby circumventing the experimental complexities associated with direct relaxation modulus measurement.

#### Relaxation Modulus Master Curves Based on Discrete and Continuous Relaxation Spectra

The discrete relaxation spectrum is presented in Table 3. The master curve of the relaxation modulus can be derived by substituting the parameters from Table 3 into Equation (7). Furthermore, in accordance with viscoelastic theory and the TTSP, the continuous relaxation spectrum enables the determination of the relaxation modulus master curve. This relationship is mathematically expressed in Equation (20), demonstrating the fundamental connection between the continuous spectral and the time-dependent relaxation modulus.

(20) $$E\left(t\right)=E_{e}+\int_{-\infty }^{\infty }H\left(\rho \right)\exp\left(-\frac{t}{\rho }\right)d\left(\ln\rho \right)$$

However, considering that the integral form of Equation (20) is complicated, the simplest trapezoidal formula in the numerical integration method is applied here to calculate the relaxation modulus. To ensure the accuracy of the calculation results, a larger integration interval (10^−30^, 10^30^) is chosen, and when this range is exceeded, the contribution of its integration result to the relaxation modulus is small and negligible. The integral Equation (20) is finally transformed into an algebraic form that is easy to solve according to the trapezoidal formula. Finally, the algebraic form is shown in Equation (21).

(21) $$E\left(t\right)\approx E_{e}+\ln10\sum_{n=1}^{n=M}\frac{H\left(\rho_{n-1}\right)\exp\left(-\frac{t}{\rho_{n-1}}\right)+H\left(\rho_{n}\right)\exp\left(-\frac{t}{\rho_{n}}\right)}{2}\Delta \mathrm{lg}\rho_{n}$$

where M denotes the number of subintervals of the integration interval (10^−30^, 10^30^), which is taken as 6000 in this paper; $\Delta \mathrm{lg}\rho_{n}$ denotes the length of each subinterval, which is taken as 0.01 in here.

Expanding Equation (21) in the form of logarithmic intervals, taking the length of each logarithmic subinterval as 0.01 and extracting the common factor, the form of the relaxation modulus expressed by algebraic operations based on the continuous relaxation time spectra can be obtained, and finally the function form of the relaxation modulus is shown in Equation (22).

(22) $$E\left(t\right)\approx E_{e}+\left(\ln10\right)\frac{0.01}{2}\left\{\begin{array}{l} \left[H\left(10^{-30}\right)\exp\left(-\frac{t}{10^{-30}}\right)+H\left(10^{-29.99}\right)\exp\left(-\frac{t}{10^{-29.99}}\right)\right]+\\ \left[H\left(10^{-29.99}\right)\exp\left(-\frac{t}{10^{-29.99}}\right)+H\left(10^{-29.98}\right)\exp\left(-\frac{t}{10^{-29.98}}\right)\right]+\ldots +\\ \left[H\left(10^{29.99}\right)\exp\left(-\frac{t}{10^{29.99}}\right)+H\left(10^{30}\right)\exp\left(-\frac{t}{10^{30}}\right)\right] \end{array}\right\}$$

**Figure 9:**
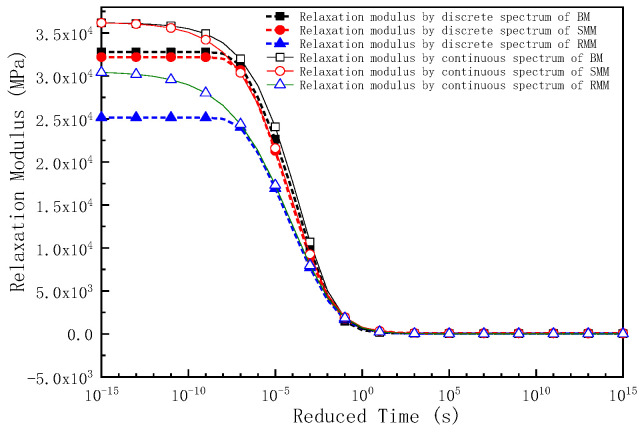
The master curve of relaxation modulus for three kinds of mixture in semi-logarithmic coordinates.

**Figure 10:**
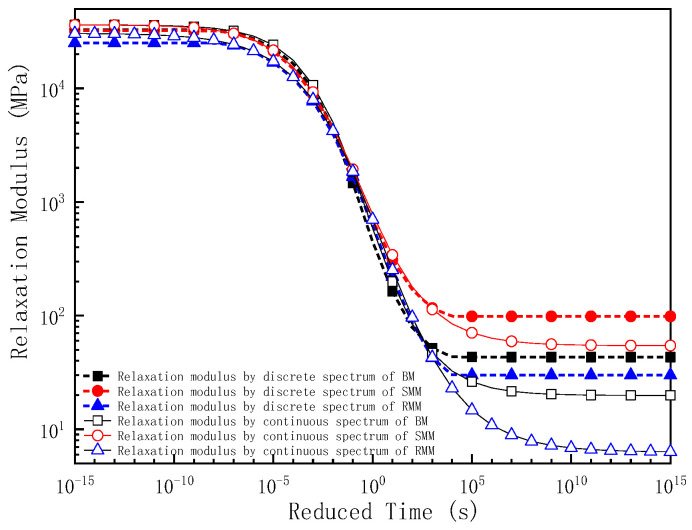
The master curve of relaxation modulus for three kinds of mixture in log–log coordinates.

As demonstrated in this study, the master curve of the relaxation modulus derived from the discrete relaxation time spectra can be effectively constructed using Equation (7). Alternatively, the continuous relaxation time spectra enable the derivation of the relaxation modulus master curve through the application of Equation (22). In this investigation, both methodologies were systematically employed to determine the relaxation modulus characteristics of the BM, SMM, and RMM at a reference temperature of 25 °C. To comparatively analyze the viscoelastic properties, the relaxation modulus results of the mixtures were plotted on semi-logarithmic coordinates (relaxation modulus vs. log relaxation time, Figure 9) and log–log coordinates (log relaxation modulus vs. log relaxation time, Figure 10).

Figure 9 and Figure 10 reveal that the relaxation modulus master curve exhibits a characteristic Z-shaped profile across an extended relaxation time domain. Specifically, the relaxation modulus asymptotically approaches its maximum value at short relaxation times, transitions to a rapid decrease in the intermediate time range, and ultimately converges to a minimum plateau at prolonged relaxation durations. This three-stage behavior aligns with the fundamental principles of polymer viscoelasticity, where short-term molecular motions dominate the initial rigidity, followed by progressive energy dissipation during intermediate time scales, and eventual equilibrium relaxation at long times. This result is consistent with the findings of many scholars [39,40]. Within the relaxation time range of 10^−7^ s to 10^5^ s, the relaxation modulus obtained from the discrete relaxation time spectra shows no significant difference compared to that derived from the continuous relaxation time spectra. However, once the relaxation time falls below 10^−7^ s, the discrete spectra yield relaxation moduli that rapidly approach larger stabilized values: 32,808 MPa for the BM, 32,196 MPa for the SMM, and 26,162 MPa for the RMM. In contrast, the continuous spectra produce relaxation moduli that gradually converge to larger stabilized values: 36,240 MPa for the BM, 36,204 MPa for the SMM, and 30,550 MPa for the RMM. When the relaxation time exceeds 10^5^ s, the discrete spectra result in relaxation moduli that sharply approach smaller stabilized values: 43 MPa for the BM, 98 MPa for the SMM, and 30 MPa for the RMM. Conversely, the continuous spectra generate relaxation moduli that progressively converge to smaller stabilized values: 20 MPa for the BM, 54 MPa for the SMM, and 6 MPa for the RMM. When the relaxation time is shorter (less than 1 s), the relaxation modulus obtained from the discrete relaxation time spectra is always smaller than that obtained from the continuous relaxation time spectra; when the relaxation time is longer (greater than 1 s), the relaxation modulus obtained from the discrete relaxation time spectra is larger than that obtained from the continuous relaxation time spectra.

From the results of the relaxation moduli of the three mixtures, it can be seen that both the relaxation modulus based on the discrete relaxation time spectra and the relaxation modulus based on the continuous time spectra reflect the same pattern; when the relaxation time is short (lower temperature), the relaxation modulus of the BM is the largest followed by the SMM, but as the relaxation time decreases further (or the temperature decreases further), the relaxation modulus of the SMM is gradually closer to that of the BM, and the relaxation modulus of the RMM is the smallest. The results indicate that in the linear viscoelastic range, the RMM has a better stress relaxation ability, and the mixture accumulates less temperature stress during the cooling process and has a better low-temperature cracking resistance; while the stress relaxation ability of the BM is very poor, it is easy for the mixture to accumulate a large thermal stress during the cooling process, thus leading to low-temperature cracking. When the relaxation time is longer (higher temperature), the relaxation modulus of the SMM is the largest followed by the BM, and the relaxation modulus of the RMM is the smallest.

### 3.4. Master Curve of Creep Compliance

Creep represents a fundamental mechanical response in viscoelastic materials, characterized by time-dependent strain accumulation under sustained constant stress. As a critical parameter for evaluating creep behavior, creep compliance serves as an essential input variable for strain prediction models in asphalt pavement design. However, the experimental determination of creep compliance at low-temperature conditions presents significant technical challenges due to the requirement of prolonged testing periods to achieve measurement accuracy. In this study, the master curves of the storage and loss compliance conforming to the approximate K-K relations were constructed from experimental dynamic modulus data. Subsequently, a simultaneous fitting procedure employing Prony series representation was implemented for both compliance components. The discrete retardation spectra were then derived through analytical transformation of the obtained Prony coefficients, ultimately enabling the determination of time-domain creep compliance master curves. This methodological framework ensures rigorous mathematical consistency between frequency-domain viscoelastic characterization and time-dependent mechanical response prediction. Furthermore, the continuous retardation spectra can be systematically determined through the inverse integral transformation of the analytical representation governing the storage compliance master curve. This derivation enables the subsequent calculation of the creep compliance master curves via numerical integration techniques applied to the continuous spectral distribution.

#### Creep Compliance Master Curve Based on Discrete and Continuous Retardation Spectra

The discrete retardation spectra are shown in Table 6, so the creep compliance master curve based on the discrete retardation spectra can be found according to Equation (12). In addition, according to viscoelastic theory and the TTSP, once obtaining the continuous retardation spectra, the creep compliance master curve based on the continuous retardation spectra can be further solved according to Equation (23).

(23) $$D\left(t\right)=D_{g}+\int_{-\infty }^{\infty }L\left(\tau \right)\left[1-\exp\left(-\frac{t}{\tau }\right)\right]d\left(\ln\tau \right)$$

However, considering the integration operation of Equation (23) is complicated, the trapezoidal formula in the numerical integration method is also applied to calculate the creep compliance. To ensure the accuracy of the calculation results, a larger integration interval (10^−30^, 10^30^) is selected, and the contribution of its integration to the creep compliance is negligible when it exceeds this range. Finally, the integral form (23) is transformed into an easy-to-operate algebraic form (24).

(24) $$D\left(t\right)\approx D_{g}+\ln10\sum_{n=1}^{n=M}\frac{L\left(\tau_{n-1}\right)\left[1-\exp\left(-\frac{t}{\tau_{n-1}}\right)\right]+L\left(\tau_{n}\right)\left[1-\exp\left(-\frac{t}{\tau_{n}}\right)\right]}{2}\Delta \mathrm{lg}\tau_{n}$$

where M denotes the number of subintervals of the integration interval (10^−30^, 10^30^), which is taken as 6000 in here; $\Delta \mathrm{lg}\tau_{n}$ denotes the length of each subinterval, which is taken as 0.01 in this article.

In order to facilitate the reader to understand, Equation (24) is expanded to the form of creep compliance based on the continuous retardation time spectra expressed by algebraic operations. Finally, the function form of the creep compliance is shown in Equation (25).

(25) $$D\left(t\right)\approx D_{g}+\left(\ln10\right)\frac{0.01}{2}\left\{\begin{array}{l} \left\{L\left(10^{-30}\right)\left[1-\exp\left(-\frac{t}{10^{-30}}\right)\right]+L\left(10^{-29.99}\right)\left[1-\exp\left(-\frac{t}{10^{-29.99}}\right)\right]\right\}+\\ \left\{L\left(10^{-29.99}\right)\left[1-\exp\left(-\frac{t}{10^{-29.99}}\right)\right]+L\left(10^{-29.98}\right)\left[1-\exp\left(-\frac{t}{10^{-29.98}}\right)\right]\right\}+\\ \ldots +\\ \left\{L\left(10^{30}\right)\left[1-\exp\left(-\frac{t}{10^{30}}\right)\right]+L\left(10^{29.99}\right)\left[1-\exp\left(-\frac{t}{10^{29.99}}\right)\right]\right\} \end{array}\right\}$$

**Figure 11:**
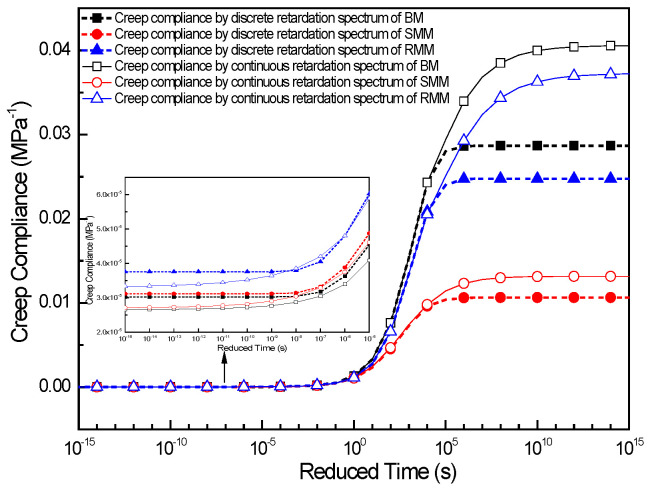
The master curve of creep compliance for three kinds of mixture.

The master curve of the creep compliance can be constructed through two distinct methodologies based on retardation time spectra analysis. Specifically, the discrete retardation time spectrum approach yields the compliance behavior through Equation (12), while the continuous retardation time spectrum formulation enables equivalent derivation via Equation (25). In this investigation, both methodologies were systematically implemented to determine the creep compliance characteristics of the BM, SMM, and RMM at a reference temperature of 25 °C. The comparative analysis of the compliance results obtained from these two spectral approaches for all three material systems is graphically presented in Figure 11, providing critical insights into their viscoelastic responses and methodological consistency.

Figure 11 reveals that the creep compliance master curve exhibits a characteristic inverted Z-shaped profile across an extended retardation time domain. Specifically, the compliance asymptotically approaches its minimum value at short retardation times, transitions to a rapid increase in the intermediate time range, and ultimately converges to a maximum plateau at prolonged relaxation durations. This result is consistent with the findings of many scholars [41,42]. Within the retardation time domain of 10^−7^ s–10^5^ s, the creep compliance derived from discrete retardation time spectra exhibits negligible deviation from that calculated by the continuous retardation time spectra. However, significant discrepancies emerge at temporal extremes. When the retardation time falls below 10^−7^ s, the discrete spectra yield creep compliance values that rapidly approach a lower asymptotic value: 3.02 × 10^−5^ MPa^−1^ for the BM, 3.11 × 10^−5^ MPa^−1^ for the SMM, and 3.76 × 10^−5^ MPa^−1^ for the RMM. In contrast, the continuous spectra gradually converge to a distinct smaller asymptotic value: 2.65 × 10^−5^ MPa^−1^ for the BM, 2.70 × 10^−5^ MPa^−1^ for the SMM, and 3.31 × 10^−5^ MPa^−1^ for the RMM, with the asymptotic values of the discrete spectra being larger than those of the continuous spectra. Conversely, when the retardation time exceeds 10^5^ s, an inverse behavioral pattern is observed: the discrete spectra result in creep compliance values that sharply approach a higher asymptotic value (2.87 × 10^−2^ MPa^−1^ for the BM, 1.07 × 10^−2^ MPa^−1^ for the SMM, and 2.48 × 10^−2^ MPa^−1^ for the RMM), while the continuous spectra progressively converge to a different larger asymptotic value (4.06 × 10^−2^ MPa^−1^ for the BM, 1.32 × 10^−2^ MPa^−1^ for the SMM, and 3.73 × 10^−2^ MPa^−1^ for the RMM), with the asymptotic values of the discrete spectra being smaller than those of the continuous spectra. These divergent characteristics fundamentally originate from the inherent mathematical distinctions between discrete summation formulations and continuous integral representations in viscoelastic modeling.

From the creep compliance results of the three mixtures, it can be acquired that both the creep compliance of the discrete retardation time spectra and the creep compliance of the continuous time spectra reflect the same pattern; when the retardation time is shorter (lower temperature), the creep compliance of the BM is the smallest, followed by the creep compliance of the SMM; however, with the further decrease in the retardation time (or temperature), the creep compliance of the SMM gradually closes to the creep compliance of the BM, and the creep compliance of the RMM is the largest; when the retardation time is longer (higher temperature), the creep compliance of the SMM is the smallest, followed by the creep compliance of the RMM, and the creep compliance of the BM is the largest. It means that in the linear viscoelastic range, the deformation of the SMM is the smallest, follow by the deformation of the RMM, and the deformation of the BM is the largest when the same load is applied at high temperature; however, the deformation of the rubber-modified asphalt mixture is the largest, followed by the deformation of the SMM, and the deformation of the BM is the smallest when the same load is applied at low temperature.

## 4. Conclusions

This study systematically investigates three asphalt mixtures (BM, SMM, RMM) by developing Generalized Sigmoidal master curve models derived from dynamic modulus testing. The developed master curves for both the storage modulus and loss modulus were verified to conform to the K-K relations, as were those for the storage compliance and loss compliance. Subsequent analyses yielded discrete and continuous relaxation spectra of the asphalt mixtures through mathematical transformation of the storage and loss modulus master curves. Concurrently, discrete and continuous retardation spectra were obtained from the storage and loss compliance master curves. Finally, the master curves of the relaxation modulus and creep compliance were successfully reconstructed using the derived discrete and continuous spectra, respectively. In addition, the relaxation and creep properties of different asphalt mixtures have been studied. The conclusions of this study are summarized as follows:

1. Both the discrete relaxation and retardation spectra demonstrate distinct bell-shaped profiles with characteristic time domains. The relaxation spectrum demonstrated a pronounced peak (maximum intensity) centered at 10^−4^ s, with the BM exhibiting the highest peak value of 7130 MPa, followed by the SMM at 6516 MPa and RMM at 4830 MPa. In contrast, the retardation spectrum demonstrated a pronounced peak (maximum intensity) centered at 10^3^ s, with the BM achieving the maximum peak value of 1.2 × 10^−2^ MPa^−1^, followed by the RMM at 9.6 × 10^−3^ MPa^−1^ and SMM at 3.2 × 10^−3^ MPa^−1^. Modified asphalt mixtures exhibit reduced relaxation spectral width/intensity and lower retardation spectral intensity compared to base mixtures, indicating enhanced stress relaxation capacity and improved deformation resistance. Notably, the SBS-modified mixture achieves optimal performance with minimal retardation spectral intensity and width, demonstrating superior deformation resistance among all mixtures. These findings quantitatively establish the viscoelastic advantages of polymer-modified asphalt binders, particularly highlighting the exceptional efficacy of SBS modification.
2. The continuous relaxation and retardation spectra also exhibit bell-shaped distributions, with the positions of the maximum peaks aligning with those of their discrete counterparts. Although the absolute intensity differs, the relative magnitude relationships among the three mixtures remain consistent with those observed in the discrete spectra. Furthermore, the retardation spectra demonstrated more pronounced asymmetry compared to their relaxation counterparts. The horizontal shift in the modified mixture’s relaxation spectrum peak toward shorter time scales indicates significantly accelerated relaxation processes relative to the BM. Comparing the continuous retardation time spectra of the BM and the modified asphalt mixtures, it revealed that the peak intensity and width of the continuous retardation spectra of the SMM were the smallest, which also indicated that the SMM has the most outstanding property to resist deformation at high temperatures.
3. The discrete and continuous relaxation spectra-derived relaxation master curves both demonstrate Z-shaped profiles with fundamental agreement, yet exhibit distinct asymptotic convergence characteristics. The discrete spectra exhibit accelerated upper asymptote convergence at short relaxation times (<10^−7^ s) with a 10–20% lower modulus maximum compared to the continuous spectra. Conversely, the discrete spectra display rapid lower asymptote attainment at extended relaxation times (>10^5^ s), maintaining minimum modulus values 1–4 times higher than those of the continuous spectra. These systematic discrepancies highlight the mathematical characterization differences between discrete and continuous spectral approaches in modeling extreme viscoelastic responses.
4. The discrete and continuous retardation spectra-derived creep compliance master curves exhibit fundamental agreement in inverted Z-shaped profiles, yet demonstrate distinct asymptotic convergence behaviors. The discrete spectra display accelerated lower asymptote attainment at short retardation times (<10^−7^ s) with 13–15% higher minimum compliance values compared to the continuous spectra. Conversely, at extended retardation times (>10^5^ s), the discrete spectra achieve rapid upper asymptote convergence while maintaining 20–35% smaller maximum compliance magnitudes than their continuous counterparts. These systematic discrepancies quantitatively reveal the mathematical characterization limitations inherent in discrete spectral analysis for modeling extreme viscoelastic responses.
5. In the linear viscoelastic range, the RMM has a better stress relaxation ability, and the mixture accumulates less thermal stress during the cooling process, thus exhibiting excellent low-temperature cracking resistance; while the BM has poor stress relaxation ability, the mixture is prone to accumulating large thermal stresses during the cooling process, resulting in low-temperature cracking. Similarly, in the linear viscoelastic range, the deformation of SMM is the smallest, followed by the RMM, and the deformation of BM is the largest when the same load is applied under high-temperature conditions. In summary, for cold regions, the application of the RMM with superior cracking resistance is recommended, while for hot climates, the SMM demonstrating enhanced rutting resistance should be preferentially adopted.

## Figures and Tables

**Table 1:** Volume parameters at optimum asphalt binder content.

Asphalt Mixture	OAC/%	Gross Density/(g·cm^−3^)	Theoretical Density/(g·cm^−3^)	Void/%	VMA/%	VFA/%	Stability/KN	Flow Value/mm
BM	4.91	2.430	2.534	4.10	13.72	70.1	9.83	3.30
SMM	5.30	2.432	2.536	4.10	14.00	70.7	11.45	3.44
RMM	5.55	2.423	2.531	4.27	14.54	70.7	10.61	3.25
Standard	—	—	—	3~5	≥13.5	65~75	≥8.0	2~4

**Table 2:** Fitting parameters for master curve construction of storage and loss modulus.

Mixture Type	Parameters								Errors/%
	$\delta^{\prime }$	$\alpha^{\prime }$	$\beta^{\prime }$	$\gamma^{\prime }$	$\lambda^{\prime }$	$C_{1}$	$C_{2}$	$k^{\prime }$	$ef_{1}$
BM	1.30	3.26	−0.40	−0.62	1.03	23.4	227.9	0.86	4.30
SMM	1.74	2.82	−0.19	−0.57	1.03	18.6	189.3	0.90	3.21
RMM	0.80	3.69	−0.70	−0.49	1.03	22.9	233.2	0.93	3.82

**Table 3:** The discrete relaxation time spectra for three kinds of mixture.

*i*	BM		SMM		RMM	
	*ρ_i_*/s	*E_i_*/MPa	*ρ_i_*/s	*ρ_i_*/s	*E_i_*/MPa	*ρ_i_*/s
1	2 × 10^−7^	2024.8	2 × 10^−7^	3467.7	2 × 10^−7^	2456.7
2	2 × 10^−6^	3867.4	2 × 10^−6^	4813.4	2 × 10^−6^	3508.5
3	2 × 10^−5^	6225.6	2 × 10^−5^	6333.8	2 × 10^−5^	4618.0
4	2 × 10^−4^	7129.2	2 × 10^−4^	6515.9	2 × 10^−4^	4830.1
5	2 × 10^−3^	6748.1	2 × 10^−3^	5567.0	2 × 10^−3^	4375.9
6	2 × 10^−2^	4096.2	2 × 10^−2^	3317.1	2 × 10^−2^	2902.5
7	2 × 10^−1^	1640.0	2 × 10^−1^	1461.0	2 × 10^−1^	1446.1
8	2	479.6	2	521.7	2	547.5
9	2 × 10^1^	148.2	2 × 10^1^	201.7	2 × 10^1^	190.8
10	2 × 10^2^	57.5	2 × 10^2^	82.6	2 × 10^2^	67.8
11	2 × 10^3^	20.5	2 × 10^3^	8.8	2 × 10^3^	11.8
12	2 × 10^4^	1.01	2 × 10^4^	4.01	2 × 10^4^	1.87
13	2 × 10^5^	0.10	2 × 10^5^	1.25	2 × 10^5^	0.62
14	2 × 10^6^	0.09	2 × 10^6^	0.10	2 × 10^6^	0.08
15	2 × 10^7^	0.0008	2 × 10^7^	0.0008	2 × 10^7^	0.0008
	*E_e_ =* 34.1		*E_e_ =* 98.4		*E_e_ =* 41.1	

**Table 4:** The error function ${ef}_{2}$ for three kinds of mixture.

Mixture Type	BM	SMM	RMM
${ef}_{2}$/%	4.9	3.5	3.5

**Table 5:** Fitting parameters for master curve construction of storage and loss compliance.

Mixture Type	Parameters								Errors/%
	$-\delta^{\prime \prime }$	$-\alpha^{\prime \prime }$	$\beta^{\prime \prime }$	$\gamma^{\prime \prime }$	$\lambda^{\prime \prime }$	$C_{1}$	$C_{2}$	$k^{\prime \prime }$	$ef_{3}$
BM	0.85	3.72	−0.62	−0.59	1.78	23.4	227.9	0.87	5.60
SMM	1.88	2.69	−0.38	−0.51	0.62	18.6	189.3	0.95	3.77
RMM	1.00	3.48	−0.78	−0.46	0.97	22.9	233.2	0.97	4.80

**Table 6:** The discrete retardation time spectra for three kinds of mixture.

*i*	BM		SMM		RMM	
	*τ_j_*/s	*D_j_*/MPa^−1^	*τ_j_*/s	*D_j_*/MPa^−1^	*τ_j_*/s	*D_j_*/MPa^−1^
1	2 × 10^−7^	2.64 × 10^−6^	2 × 10^−7^	4.09 × 10^−6^	2 × 10^−7^	6.11 × 10^−6^
2	2 × 10^−6^	6.98 × 10^−6^	2 × 10^−6^	7.29 × 10^−6^	2 × 10^−6^	8.06 × 10^−6^
3	2 × 10^−5^	9.69 × 10^−6^	2 × 10^−5^	1.15 × 10^−5^	2 × 10^−5^	1.55 × 10^−5^
4	2 × 10^−4^	3.01 × 10^−5^	2 × 10^−4^	2.80 × 10^−5^	2 × 10^−4^	3.14 × 10^−5^
5	2 × 10^−3^	5.26 × 10^−5^	2 × 10^−3^	5.34 × 10^−5^	2 × 10^−3^	5.90 × 10^−5^
6	2 × 10^−2^	1.51 × 10^−4^	2 × 10^−2^	1.37 × 10^−4^	2 × 10^−2^	1.38 × 10^−4^
7	2 × 10^−1^	4.37 × 10^−4^	2 × 10^−1^	3.59 × 10^−4^	2 × 10^−1^	3.54 × 10^−4^
8	2 × 10^0^	1.09 × 10^−3^	2 × 10^0^	8.74 × 10^−4^	2 × 10^0^	8.57 × 10^−3^
9	2 × 10^1^	2.91 × 10^−3^	2 × 10^1^	1.81 × 10^−3^	2 × 10^1^	2.43 × 10^−3^
10	2 × 10^2^	5.94 × 10^−3^	2 × 10^2^	2.73 × 10^−3^	2 × 10^2^	5.58 × 10^−3^
11	2 × 10^3^	1.15 × 10^−2^	2 × 10^3^	3.21 × 10^−3^	2 × 10^3^	9.59 × 10^−3^
12	2 × 10^4^	5.44 × 10^−3^	2 × 10^4^	1.81 × 10^−3^	2 × 10^4^	5.41 × 10^−3^
13	2 × 10^5^	1.01 × 10^−3^	2 × 10^5^	7.27 × 10^−4^	2 × 10^5^	2.13 × 10^−3^
14	2 × 10^6^	4.03 × 10^−3^	2 × 10^6^	2.02 × 10^−4^	2 × 10^6^	5.79 × 10^−4^
15	2 × 10^7^	1.01 × 10^−5^	2 × 10^7^	1.01 × 10^−5^	2 × 10^7^	5.19 × 10^−6^
	*D_g_ =* 3.43 × 10^−5^		*D_g_ =* 3.07 × 10^−5^		*D_g_ =* 3.76 × 10^−5^	

**Table 7:** The error function $ef_{4}$ for three kinds of mixture.

Mixture Type	BM	SMM	RMM
$ef_{4}$/%	7.9	5.6	3.7

## Data Availability

Data are contained within the article.

## Abbreviations

The following abbreviations are used in this manuscript:

K-K relations	Kramers–Kronig Relations
STEM	Scanning tunneling electron microscopy
ZSV	Zero-shear viscosity
BM	Base asphalt mixture
SMM	SBS-modified asphalt mixture
RMM	Crumb rubber-modified asphalt mixture
TTSP	Time–temperature superposition principle
WLF	Williams–Landel–Ferry
SBS	Styrene–butadiene–styrene

## Appendix A

### Appendix A.1. Calculation Equations for Dynamic Modulus and Phase Angle

The dynamic modulus represents the stiffness of the material under cyclic loading and the phase angle represents quantifying the time-dependent viscous response relative to the elastic behavior. The calculation equations for complex modulus $E^{*}$, dynamic modulus $\left|E^{*}\right|$, and phase angle φ are provided in Equations (A1)–(A3)

(A1) $$E^{*}=\frac{\sigma \left(t\right)}{\varepsilon \left(t\right)}=\frac{\sigma_{0}e^{i\left(\omega t+\varphi \right)}}{\varepsilon_{0}e^{i\left(\omega t\right)}}=\frac{\sigma_{0}}{\varepsilon_{0}}e^{i\varphi }$$

(A2) $$\left|E^{*}\right|=\frac{\sigma_{0}}{\varepsilon_{0}}$$

(A3) $$\varphi =\frac{t_{i}}{t_{p}}360$$

where $E^{*}$ denotes the complex modulus, MPa; $\sigma_{0}$ denotes the peak stress, MPa; $\varepsilon_{0}$ denotes the peak strain; ω denotes the angular frequency, rad/s; $\left|E^{*}\right|$ denotes the dynamic modulus, MPa; φ denotes the phase angle, °; $t_{i}$ denotes the average lag time of the peak stress and peak strain in the last five loading cycles, s; $t_{p}$ denotes the average loading period of the last five loading cycles, s.

### Appendix A.2. Calculation Equations for Storage Modulus and Loss Modulus

The storage modulus $E^{\prime }\left(w\right)$ and loss modulus $E^{\prime \prime }\left(w\right)$ can be derived from the experimentally determined dynamic modulus $\left|E^{*}\right|$ through the fundamental relationships expressed in Equations (A4) and (A5):

(A4) $$E^{\prime }\left(w\right)=\frac{\sigma_{0}}{\varepsilon_{0}}\cos\left(\varphi \right)=\left|E^{*}\left(w\right)\right|\cos\left(\varphi \right)$$

(A5) $$E^{\prime \prime }\left(w\right)=\frac{\sigma_{0}}{\varepsilon_{0}}\sin\left(\varphi \right)=\left|E^{*}\left(w\right)\right|\sin\left(\varphi \right)$$

### Appendix A.3. Calculation Equations for Storage Compliance and Loss Compliance

Based on the experimental test results of the dynamic modulus, the calculation results of the storage compliance $D^{\prime }\left(w\right)$ and loss compliance $D^{\prime \prime }\left(w\right)$ can also be further obtained. The calculation forms are shown in the following Equations (A6) and (A7), respectively.

(A6) $$D^{\prime }\left(w\right)=\frac{E^{\prime }\left(w\right)}{{\left[E^{\prime }\left(w\right)\right]}^{2}+{\left[E^{\prime \prime }\left(w\right)\right]}^{2}}=\frac{\cos\left(\varphi \right)}{\left|E^{*}\left(w\right)\right|}$$

(A7) $$D^{\prime \prime }\left(w\right)=\frac{E^{\prime \prime }\left(w\right)}{{\left[E^{\prime }\left(w\right)\right]}^{2}+{\left[E^{\prime \prime }\left(w\right)\right]}^{2}}=\frac{\sin\left(\varphi \right)}{\left|E^{*}\left(w\right)\right|}$$

### Appendix A.4. Relationship Between Storage Modulus Master Curves and Continuous Relaxation Time Spectra

To enable direct derivation of the continuous relaxation time spectra from the storage modulus master curves, the analytical expression (Equation (2)) is rewritten into the mathematical framework defined in Equation (A8).

(A8) $$E^{\prime }\left(w\right)=\exp\left(A\right)\exp\left(\frac{B}{{\left[1+\lambda^{\prime }\exp\left(C+D\ln\alpha_{T}+D\ln w\right)\right]}^{\frac{1}{\lambda^{\prime }}}}\right)$$

where $A=\delta^{\prime }\ln10$; $B=\alpha^{\prime }\ln10$; $C=\beta^{\prime }-\gamma^{\prime }\log\left(2\pi \right)$; $D=\gamma^{\prime }/\ln10$.

The continuous relaxation spectra model characterized by the parametric form of the master curve of the storage modulus can be derived by substituting Equation (24) into Equation (23) and further simplifying it according to Euler’s formula, and the form of the finalized continuous relaxation spectra is as in Equation (A9).

(A9) Hρ=±2πexpAImexpPρλ

where $H\left(\rho \right)$ denotes the continuous relaxation time spectra; $P\left(\rho \right)$ denotes the function on $X\left(\rho \right)$ and $Y\left(\rho \right)$; both $X\left(\rho \right)$ and $Y\left(\rho \right)$ are described in the functional form of *B*, *C*, and *D*; the derived mathematical formulations for $P\left(\rho \right)$, $X\left(\rho \right)$, and $Y\left(\rho \right)$ are ultimately expressed through the analytical relationships presented in Equations (A10), (A11), and (A12), respectively.

(A10) $$P\left(\rho \right)=X\left(\rho \right)\mp Y\left(\rho \right)$$

(A11) $$X\left(\rho \right)=\frac{B^{\lambda^{\prime }}\left[1+\lambda^{\prime }\exp\left(E\right)\cos\frac{\pi D}{2}\right]}{1+{\left(\lambda^{\prime }\right)}^{2}\exp\left(2E\right)+2\lambda^{\prime }\exp\left(E\right)\cos\frac{\pi D}{2}}$$

(A12) $$Y\left(\rho \right)=\frac{B^{\lambda^{\prime }}\left[\lambda^{\prime }\exp\left(E\right)\sin\frac{\pi D}{2}\right]}{1+{\left(\lambda^{\prime }\right)}^{2}\exp\left(2E\right)+2\lambda^{\prime }\exp\left(E\right)\cos\frac{\pi D}{2}}$$

where $E=C+D\ln\frac{\alpha_{T}}{\rho }$.

To further simplify the form for the continuous relaxation spectra in Equation (A9), it can be assumed that $F\left(\rho \right)=X{\left(\rho \right)}^{2}+Y{\left(\rho \right)}^{2}$;$G\left(\rho \right)=\arctan\left[Y\left(\rho \right)/X\left(\rho \right)\right]$; then, Equation (A9) can again be rewritten in the functional form of Equation (A13).

(A13) $$H\left(\rho \right)=-\left(\frac{2}{\pi }\right)\exp\left(A+F^{\frac{1}{2\lambda^{\prime }}}\cos\frac{G}{\lambda^{\prime }}\right)\sin\left(F^{\frac{1}{2\lambda^{\prime }}}\sin\frac{G}{\lambda^{\prime }}\right)$$

The resulting analytical formulation corresponding to the continuous relaxation time spectrum is expressed through Equation (A13), derived through integration of the Generalized Sigmoidal storage modulus master curve with Euler’s formula. This unified representation systematically incorporates parameters ($\delta^{\prime }$, $\alpha^{\prime }$, $\beta^{\prime }$, $\gamma^{\prime }$, $\lambda^{\prime }$) from both the storage modulus master curve model and the loss modulus master curve model, ensuring constitutive consistency across viscoelastic property characterization. Finally, the continuous relaxation time spectra in the reference temperature of 25 °C are obtained by substituting the model parameters in Table 2 into Equation (A13). In particular, when $\lambda^{\prime }=1$, Equation (A13) can be transformed into the continuous relaxation time spectra expressed in terms of the Sigmoidal master curve model parameters, which is described in the form of Equation (A14). In addition, the Equation (A14) in Appendix A.4. is Equation (17) in the main body of the manuscript.

(A14) $$H\left(\rho \right)=-\left(\frac{2}{\pi }\right)\exp\left(A+X\left(\rho \right)\right)\sin\left(Y\left(\rho \right)\right)$$

### Appendix A.5. Relationship Between Storage Compliance Master Curves and Continuous Retradation Time Spectra

Similar to the derivation of the continuous relaxation time spectrum, to enable direct derivation of the continuous retardation time spectra from the storage compliance master curves, the analytical expression (Equation (4)) is rewritten into the mathematical framework defined in Equation (A15).

(A15) $$D^{\prime }\left(w\right)=\exp\left(A^{\prime }\right)\exp\left(\frac{B^{\prime }}{{\left[1+\lambda^{\prime \prime }\exp\left(C^{\prime }+D^{\prime }\ln\alpha_{T}+D^{\prime }\ln w\right)\right]}^{\frac{1}{\lambda^{\prime \prime }}}}\right)$$

where $A^{\prime }=\delta^{\prime \prime }\ln10$; $B^{\prime }=\alpha^{\prime \prime }\ln10$; $C^{\prime }=\beta^{\prime \prime }-\gamma^{\prime \prime }\log\left(2\pi \right)$; $D^{\prime }=\gamma^{\prime \prime }/\ln10$.

Through the substitution of Equation (A15) into Equation (18) and the application of Euler’s formula, the continuous retardation spectra defined by the functional formalism of the storage compliance master curve can be mathematically derived. The resulting expression for the continuous relaxation spectra is then obtained as Equation (A16).

(A16) Lτ=∓2πexpA′ImexpPτλ″

where $L\left(\tau \right)$ denotes the continuous retardation time spectra; $P\left(\tau \right)$ can be described in the functional form of $X\left(\tau \right)$ and $Y\left(\tau \right)$; and both $X\left(\tau \right)$ and $Y\left(\tau \right)$ can be described as the function of $B^{\prime }$; $C^{\prime }$; $D^{\prime }$; and finally, the functional forms $P\left(\tau \right)$, $X\left(\tau \right)$, and $Y\left(\tau \right)$ are shown in Equations (A17)–(A19).

(A17) $$P\left(\tau \right)=X\left(\tau \right)\mp Y\left(\tau \right)$$

(A18) $$X\left(\tau \right)=\frac{B^{\lambda^{\prime \prime }}\left[1+\lambda^{\prime \prime }\exp\left(E^{\prime }\right)\cos\frac{\pi D^{\prime }}{2}\right]}{1+{\left(\lambda^{\prime \prime }\right)}^{2}\exp\left(2E^{\prime }\right)+2\lambda^{\prime \prime }\exp\left(E^{\prime }\right)\cos\frac{\pi D^{\prime }}{2}}$$

(A19) $$Y\left(\tau \right)=\frac{B^{\lambda^{\prime \prime }}\left[\lambda^{\prime \prime }\exp\left(E^{\prime }\right)\sin\frac{\pi D^{\prime }}{2}\right]}{1+{\left(\lambda^{\prime \prime }\right)}^{2}\exp\left(2E^{\prime }\right)+2\lambda^{\prime \prime }\exp\left(E^{\prime }\right)\cos\frac{\pi D^{\prime }}{2}}$$

where $E^{\prime }=C^{\prime }+D^{\prime }\ln\frac{\alpha_{T}}{\tau }$.

To further simplify the form of the continuous retardation spectra in Equation (A16), assume $F\left(\tau \right)=X{\left(\tau \right)}^{2}+Y{\left(\tau \right)}^{2}$, $G\left(\tau \right)=\arctan\left[Y\left(\tau \right)/X\left(\tau \right)\right]$; then, the Equation (A16) can again be described in the form of Equation (A20).

(A20) $$L\left(\tau \right)=-\left(\frac{2}{\pi }\right)\exp\left(A^{\prime }+F^{\frac{1}{2\lambda^{\prime \prime }}}\cos\frac{G}{\lambda^{\prime \prime }}\right)\sin\left(F^{\frac{1}{2\lambda^{\prime \prime }}}\sin\frac{G}{\lambda^{\prime \prime }}\right)$$

Equation (A20) is the functional form for the continuous retardation time spectra constructed based on the Generalized Sigmoidal storage compliance master curve model and Euler’s formula, which shares parameters with the storage compliance master curve and the loss compliance master curve ($\delta^{\prime \prime }$, $\alpha^{\prime \prime }$, $\beta^{\prime \prime }$, $\gamma^{\prime \prime }$, $\lambda^{\prime \prime }$). Finally, the continuous retardation time spectra in the reference temperature of 25 °C are obtained by substituting the model parameters in Table 5 into Equation (A20). In particular, when $\lambda^{\prime }=1$, Equation (A20) can be transformed into the continuous retardation time spectra expressed in terms of the Sigmoidal master curve model parameters, whose form is described in Equation (A21). In addition, the Equation (A21) in Appendix A.5 is Equation (17) in the main body of the manuscript.

(A21) $$L\left(\tau \right)=\left(\frac{2}{\pi }\right)\exp\left[A-X\left(\tau \right)\right]\sin\left[Y\left(\tau \right)\right]$$
